# Inferring DNA Kinkability from Biased MD Simulations

**DOI:** 10.1021/acs.jctc.5c01660

**Published:** 2026-01-13

**Authors:** Arianna Fassino, Enrico Carlon, Aderik Voorspoels

**Affiliations:** † 54517 Soft Matter and Biophysics, KU Leuven, Celestijnenlaan 200D, Leuven B-3001 Belgium; ‡ Institute of Systems, Molecular and Integrative Biology, University of Liverpool, Liverpool L69 7ZB, U.K.

## Abstract

In several biological processes, such as looping, supercoiling, and DNA–protein interactions, DNA is subject to very strong deformations. While coarse-grained models often approximate DNA as a smoothly bendable polymer, experimental and theoretical studies have demonstrated that mechanical stress can induce localized kinks. Here, we employ the Rigid Base Biasing of Nucleic Acids (RBB-NA) algorithm to systematically probe the properties of highly deformed DNA in all-atom simulations of short dodecamers. A simultaneous bias in bending (roll) and twist is applied locally to two consecutive base pairs in the center of the dodecamers. Using umbrella sampling, we construct free energy landscapes that reveal sequence-dependent effects for kink formation and quantify the energetic cost of kinking. We identify distinct features in the free energy profiles highlighting anharmonic effects, such as asymmetries in the positive vs negative roll. Our analysis suggests two distinct kink types characterized either by positive roll and undertwisting (twist-bend kinks) or by negative roll without excess twist (pure bend kinks). The former are frequently observed in DNA–protein structures and are expected to be favored in vivo in negatively supercoiled chromosomes. The latter has been observed in DNA simulations of minicircles and is favored in torsionally constrained DNA.

## Graphical abstract

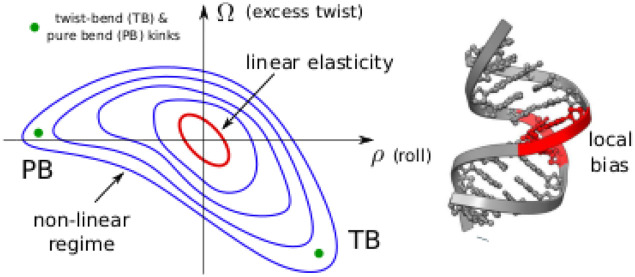

## Introduction

Fifty years ago, in a paper entitled ″The Kinky Helix″,^1^ Crick and Klug proposed a geometrical model that incorporated sharp bends in DNA, or DNA kinks, to explain the folded structure of chromatin in the cell nuclei. They suggested that kinks could form in DNA without disrupting base pairing but through the unstacking of two consecutive base pairs. According to their model, these kinks would be localized at two specific consecutive base pairs with “bond lengths and dihedral angles assuming chemically acceptable values″,^1^ while the double helix would maintain its ordinary B-form at the two sides of the kink. Although sharp kinks are not essential to explain the primary features of chromatin structure—where DNA is uniformly wrapped around histone proteins—Crick and Klug’s paper sparked interest in the mechanical properties of the DNA double helix and the possibility of significant conformational changes at the base pair level.

### Experimental Observations of Kinks

Sharp kinks were indeed experimentally observed in several protein–DNA complexes.^2–4^ Anomalously high bendability of bare DNA molecules, possibly associated with the presence of kinks, was reported in DNA cyclization experiments,^5^ atomic force microscopy (AFM),^6^ DNA minicircles,^7^ and fluorescence resonance energy transfer (FRET).^8,9^ More recently, X-ray and neutron small-angle scattering measurements suggested the presence of kinks in free linear DNA in solution.^10^ Although some of the experimental procedures have been criticized by some authors,^11–13^ there is nowadays a general consensus on experimental evidence for the very high bendability of short DNA sequences.^14^ This bendability goes well beyond the limit expected from standard elastic models, such as the twistable wormlike chain.^15^

### Kinks in MD Simulations

Sharp kinks of various structural conformations were also observed in all-atom simulations of small DNA minicircles,^16–18^ highly bent short linear DNA,^19^ or torsionally constrained molecules.^20^ These studies revealed different structural properties of the kinks. In one of the earliest simulation studies, Lankaš et al.^16^ observed two types of kinks, referred to as Type I and Type II kinks. Type I kinks closely resembled the structure proposed by Crick and Klug^1^ and had intact base pairing, while Type II kinks involved a single base pair disruption. Different types of kinks were also observed in simulations of supercoiled minicircles,^17,18^ which used an updated force field, different from that used in ref 16. Simulations of linear DNA, biased to assume a strong bend by an applied constraint at the two ends of the molecule, found evidence of the presence of sharp kinks,^19^ predominantly of Type II. Bending free energies were estimated from umbrella sampling techniques.^19^

### Models of Kinkable DNA

In parallel, several groups developed and studied various analytical models that account for anomalous DNA bendability.^12,15,21–23^ To account for sharp kinks observed in AFM experiments, some authors invoked a generic bending energy model:^12,15^

1 $$\beta E=\underset{n}{\sum }g\left(\theta_{n}\right)$$

with β = 1/*k*_B_ *T* and *g*(θ) describing the energy dependence on a local bending angle θ_*n*_. The total energy is then obtained by summing these local terms along the sequence. In these models, the sequence is discretized into segments comprising a few helix turns; hence, the sum over *n* can refer to a single base-pair step or to a stretch of ∼10–20 bases. Various forms for *g*(θ) were considered as an alternative to the WLC harmonic elasticity *g*(θ) ∝ θ^2^. We mention here the examples of the linear subelastic chain^6^ *g*(θ) = α|θ| and the hinge model:

2 $$g\left(\theta \right)=\min\left(\frac{g_{1}}{2}\theta^{2},h+\frac{g_{2}}{2}{\left(\theta -\theta_{0}\right)}^{c}\right)$$

where *h* is a parameter defining the energetic cost of a kink. Two choices of exponent *c* were considered: *c* = 2 in.^22^ and *c* = 6 in.^12^ The hinge model describes a kinked conformation as a second local energy minimum on θ_0_, while small bending deformations (small θ ≪ θ_0_) would be described by a harmonic term *g*_1_θ^2^/2. Yan and Marko^21^ described kinks as locally disrupted base pairing causing very flexible single-stranded DNA stretches, which would favor sharp DNA bending. In their model, the bending energy contains an additional Boolean variable σ; hence it is given by a function *g*(θ, σ), fluctuating between the B-DNA (σ = 0) and locally melted, kinked (σ = 1) state. This was modeled as *g*(θ, 0) = *a*θ^2^ and *g*(θ, 0) = *a*′θ^2^ + μ, with *a*′ ≪ *a* and μ the free energy associated with the creation of a small melting bubble.^21^ Although simple functions for *g*(θ) may be useful to provide an analytical model to estimate the effect of kinks, it is desirable to gain some more insights on anharmonic effects directly from detailed MD simulations, which is the aim of this paper.

While eq 1 uses a single bending angle, at the base pair level, there are two bending directions defined as tilt and roll. Together with twist, these describe the major deformation modes of the double helix. In the harmonic approximation, the DNA energy using tilt, roll, and excess twist (τ, ρ, Ω) variables is given by *E* = ∑_*n*_ ε_*n*_, where the sum is over dinucleotide steps and with

3 $$\beta \varepsilon_{n}=\frac{1}{2}\left[A_{n}^{\tau }\tau_{n}^{2}+A_{n}^{\rho }\rho_{n}^{2}+C_{n}\Omega_{n}^{2}+2G_{n}\Omega_{n}\rho_{n}\right]$$

The stiffnesses ( $A_{n}^{\tau }$ , $A_{n}^{\rho }$ , *C*_*n*_, *G*_*n*_) are sequence-dependent, as indicated by their *n*-dependence. The predominant off-diagonal interaction is the coupling *G*_*n*_ between roll and twist.^24^ Various effects of this coupling have been discussed in the recent literature.^25,26^ A tilt deformation is much stiffer than roll $\left(A_{n}^{\tau }\gg A_{n}^{\rho }\right)$ ; therefore, large bending deformations predominantly involve roll. We also note that the parameters τ, ρ, and Ω have off-site couplings via quadratic terms, as, e.g., τ_*n*_τ_*n* + 1_.^27–29^ However, these couplings are found to be very weak, especially for roll,^30,31^ and will be neglected in the theoretical description of this paper. The presence of the twist–roll coupling suggests that a large roll should involve a twist deformation as well, consistent with observations from simulations.^25,26^ We will seek to infer the structure of the free energy (eq 3) beyond the quadratic approximation.

### Kinks in Protein–DNA Complexes: The IHF

In 1998, Olson et al.^34^ developed a method to extract elastic parameters of DNA deformations from high-resolution protein–DNA crystal structures. They modeled DNA as a harmonic system (as eq 3) and obtained sequence-dependent stiffness matrices from these structural data. Following the same line of thought, one can infer the kinkability of bare DNA from highly bent DNA bound to proteins. A particularly interesting case for highly bent conformations is that of the Integration Host Factor (IHF), a DNA-binding protein found in bacteria that plays an important role in chromosomal compaction and gene regulation. IHF is a heterodimer protein consisting of two subunits, which induce two sharp bends in DNA. The DNA-IHF also exhibits complex kinetics, which were studied in detail in refs 35 and 36. Figure 1 shows (a) a structure of the IHF-DNA complex from the Protein Data Bank and (b) the corresponding values of the coordinates tilt, roll, and twist as calculated by the web3dna server^33^ from the PDB file of the crystal structure. The two kinks are observed in TT and AA steps, with the latter intentionally nicked to facilitate crystallization of the sample.^32^ The tilt, roll, and twist parameters around the nicked site are not shown, as the nick breaks the B-DNA structure, and such parameters do not provide useful information about the DNA conformation. The TT kink shows a strong increase in the roll (+60°) and a more moderate decrease in the twist (−10°), as expected from a positive twist–roll coupling (several simulation studies^30,37^ have shown that *G* > 0). At the kink site, there is a modest increase in tilt (+5°), suggesting that one can indeed neglect this stiff degree of freedom in analyzing DNA kinks. We will focus here on deformations involving roll and twist, as these are the most relevant parameters to describe kinks.^34^ Sharp kinks in DNA–protein complexes are often characterized by a strong positive roll and undertwist; see Supporting Information for some more examples. In general, in protein–DNA complexes the binding affinity of a given sequence to a protein is driven by the DNA chemical structure (direct readout), as well as by the propensity of the sequence to assume conformations that facilitate that binding (indirect readout).^38^ It is therefore interesting to explore the propensity to kink for DNA sequences that form sharp kinks when binding to proteins.

**Figure 1:**
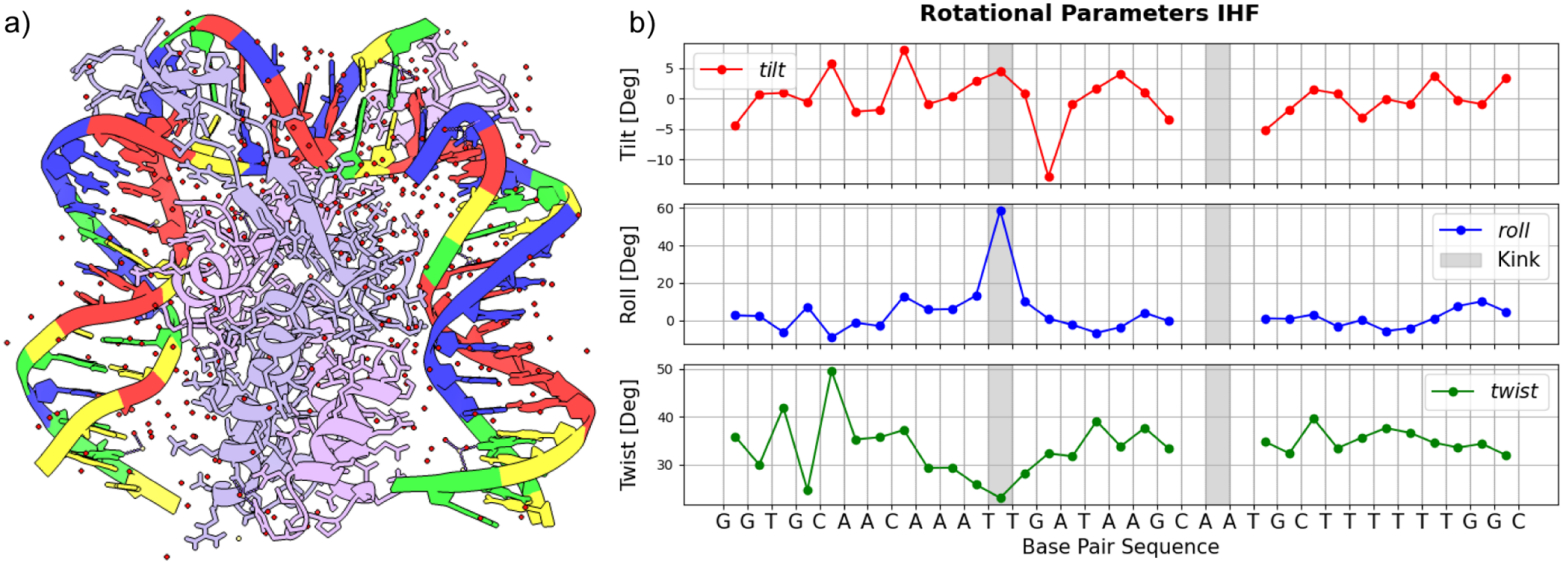
a) Crystal structure of an IHF-DNA complex from the Protein Data Bank^32^ visualized via ChimeraX. The DNA forms two sharp kinks, resulting in a global bend angle of ∼180° over the full range of the sequence. b) Plots of Tilt, Roll, and Twist angles per base pair as obtained from the W3DNA web server.^33^ The gray areas indicate the kinks’ location. As the DNA is nicked in the AA step (to favor crystallization), the rotational parameters are not shown. The TT-kink is characterized by a strong increase in the roll and a more moderate decrease in the twist, as expected from a positive twist–roll coupling.

### Kinkability from Biased MD Simulations

We explore here the tendency of DNA molecules to kink using all-atom MD simulations and focus on the behavior of the roll and twist coordinates in regimes beyond the quadratic approximation (eq 3). In unbiased MD simulations, it is not possible to observe the spontaneous formation of kinks due to limitations in the time scale currently reachable in all-atom simulations. Prior MD simulations explored the kinkability of DNA minicircles,^16–18^ which are already bent in their initial conformation. Alternatively, strong bending deformations were induced by biasing the two ends of a 15-bp linear DNA molecule.^19^ In both setups, kinks were seen at more deformable sites along the sequence and were sometimes not localized, as curvature or over- and undertwisting involved several consecutive base pairs. Other MD studies showed more localized kinks by simulating the entire IHF-DNA complex.^36^ This approach is crucial for capturing the interplay between protein-binding mechanisms and DNA kinkability, but it cannot disentangle the two.

Here we used RBB-NA,^39^ a recently developed algorithm that can bias any of the 12 rigid base coordinates (or any combination thereof) at a local scale: either at a specific site or at multiple sites. By biasing both roll and twist at a specific DNA site, we infer the tendency of bare DNA to take on highly deformed conformations via the calculation of two-dimensional free energy landscapes as a function of local roll and twist coordinates. The results show that kinkability is strongly sequence dependent, with different sequences showing quite different behaviors. Some sequences are highly deformable and can accommodate strong and positive (roll) angles with a modest undertwist. This happens specifically for the IHF-bound DNA sequence at the kink site, showing an inflection (although not a metastable local minimum) in the free energy landscape corresponding to values close to those observed in crystal structures, as shown in Figure 1(b). Other sequences, however, are stiffer, with a free energy increasing much more rapidly as a function of roll and twist angles. In addition, we find a strongly asymmetric behavior of DNA at positive and negative rolls.

## Materials and Methods

### Sequences Used

Table 1 shows the six dodecamers analyzed in this work. A bias on roll and twist was applied between the two central nucleotides (in bold) of all sequences. Sequences 1 and 2 bind to the IHF protein, with sharp kinks forming in the central TT and AA pairs. These two sequences are overlapping parts of the DNA of the IHF-DNA complex studied in ref 32 and shown in Figure 1. Sequence 3 is part of the Hbb-DNA complex, which has kinks structurally similar to those of the IHF (see ref 40 and Supporting Information). Histone-like protein from *Borrelia burgdorferi* (Hbb in short) is a protein which, like IHF, induces sharp bends on DNA with large positive roll and undertwist at the two binding sites (the Supporting Information reports the rotational parameters for the DNA-Hbb complex from the crystal structure data^40^). Unlike IHF, which has a high affinity for specific consensus sequences, Hbb binds DNA nonspecifically, preferring binding to A-T rich regions, which are generally more flexible.^40^ Sequence 4 is the Drew–Dickerson Dodecamer (DDD), a well-known sequence that has been widely studied in structural DNA investigations. Sequences 5 and 6 replicate Seq 1 and Seq 3, respectively, but with changes in the two biased central nucleotides, with CC and GC replacing the original TT and AT.

**Table 1:** List of Dodecamer Sequences Simulated^a^

Name	Origin	Sequence
seq1	IHF bind.	ACAAA **TT** GATAA
seq2	IHF bind.	TAAGC **AA** TGCTT
seq3	Hbb bind.	ATACT **AT** ATGTC
seq4	DDD	CGCGA **AT** TCGCG
seq5	IHF mut.	ACAAA **CC** GATAA
seq6	Hbb mut.	ATACT **GC** ATGTC

a A bias on roll and twist is applied between the central nucleotides shown in bold to obtain free energies via umbrella sampling. Sequences 1 and 2 are IHF-binding sequences centered around the two kink locations; see Figure 1. Sequence 3 binds to Hbb, the Histone-like protein from the bacterium *Borrelia burgdorferi*. Sequence 4 is Drew–Dickinson Dodecamer (DDD). Sequence 5 is the same as Seq 1, with a CC pair replacing the central TT pair. Sequence 6 is mutated from the Hbb-binding sequence (Seq 3) mutated, with GC replacing the original AT central base step, instead of the natural AT step. Sequences 1–3 form sharp kinks when bound to proteins.

The limited set of sequences examined in this study does not clearly span all of the possible nucleotide combinations. Over the past decade, unbiased simulations have demonstrated that DNA mechanics depends not only on the identity of the central base pair step but also on the surrounding nucleotides.^41^ Building on this, a consortium of several laboratories conducted a comprehensive and systematic investigation of the flexibility of the complete set of 136 distinct tetranucleotides.^41^ It was found that certain nucleotide combinations (notably CG steps) display a complex polymorphic behavior,^42^ characterized by transitions between high-twist and low-twist conformations that equilibrate only on very long time scales (on the order of ∼100 ns), which lies well beyond the reach of our biasing scheme. In this work, we concentrate on the high-deformation regime for a limited set of sequences (excluding polymorphic CG steps), while keeping in mind that our description of this regime is still far from exhaustive. We calculated, for all six sequences, the free energy Δ*F*(ρ, Ω) as a function of the roll (ρ) and twist (Ω) deformations of the two central base pairs. The protocol is schematically illustrated in Figure 2 and explained in detail in the following sections.

**Figure 2:**
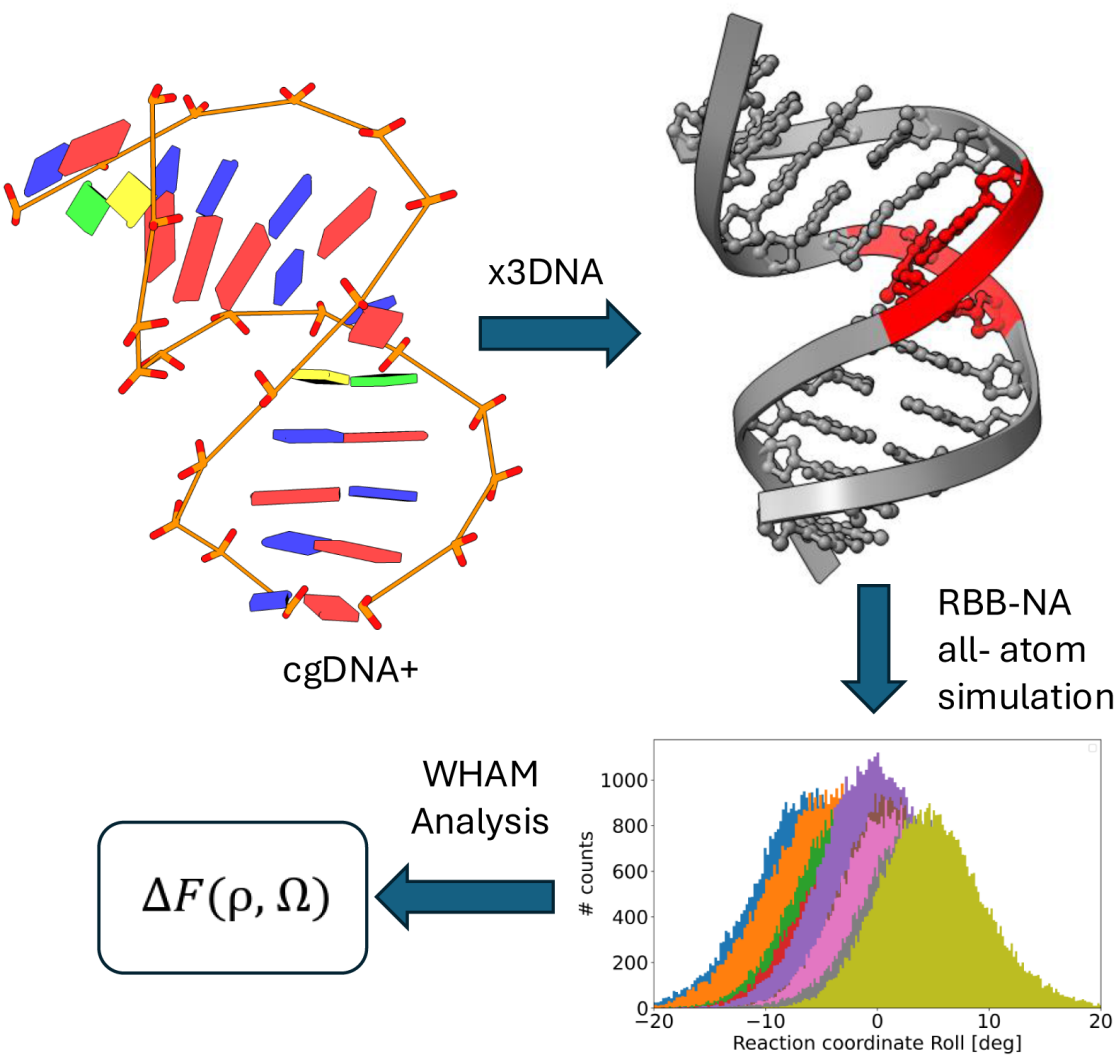
A schematic representation of the different steps followed for the calculation of the free energy Δ*F*(ρ, Ω). (1) The coarse-grained cgDNA+ model is biased to produce a twisted and bent conformation. (2) The all-atom structure is then obtained by the x3DNA software. (3) Such conformations are further sampled with the RBB-NA algorithm, which keeps the biases on the central nucleotides. (4) The WHAM analysis collects the data from the different biases to reconstruct the unbiased free energy.

### Generating Input Configurations via cgDNA+ and x3DNA

cgDNA+ is a coarse-grained model developed to simulate sequence-dependent mechanical and structural properties of double-stranded DNA.^43–46^ The standard cgDNA model^43^ parametrizes the DNA conformations using the canonical 12 coordinates defined by the Tsukuba convention. Six of these are intra-base pair coordinates (buckle, propeller, opening, shear, stretch, stagger), while the remaining six are inter-base pair coordinates (tilt, roll, twist, shift, slide, rise). In cgDNA+, the upgraded version,^44,45^ the phosphate groups are modeled explicitly, which leads to 24 coordinates per bp. cgDNA and cgDNA+ sample the conformational spaces using a Monte Carlo algorithm with a quadratic free energy model for the coarse-grained coordinates, similar to eq 3 but with coupling extending to further neighbors (as τ_*n*_τ_*n* +*k*_ with *n* > 0). The cgDNA+ shows an excellent agreement with all-atom data, capturing some next-neighbor correlations that are missed by the earlier cgDNA model.^47^ This agreement holds in the small deformation regime, where bending and twist angles are small, and the deformation free energy is well-approximated by a quadratic function. In this work, we used cgDNA+ for two purposes: (1) for the calculation of free energies in the high deformation regime and (2) to generate highly deformed atomic structures (via the x3DNA software) used as input configurations for the biased all-atom simulations. Step (1) produces quadratic free energies but also serves as a test for the biasing scheme and the reweighting method, which is similar to that used in all-atom simulations. Step (2) produces input configurations that are locally deformed and are expected to be close to those later generated during the biased all-atom RBB-NA runs. This reduces the equilibration time and makes the subsequent free energy sampling more efficient.

The biasing potential was chosen in the form:

4 $$V=\frac{K}{2}\left[{\left(\rho -\mathrm{\rho ̅}\right)}^{2}+{\left(\Omega -\gamma \mathrm{\rho ̅}\right)}^{2}\right]$$

with ρ the roll and Ω the excess twist, as in eq 3. For simplicity, we used the same stiffness *K* for twist and roll, although different combinations are possible. For cgDNA+ simulations, the value was set to *K* = 1000 kJ/mol. The same type of bias was also employed for RBB-NA all-atom simulations, but with a smaller *K*, as discussed below. We explored a grid of possible values of roll and excess twist, ρ̅ nd Ω̅ = γρ̅. Both in cgDNA+ and all-atom simulations, biases on roll and twist were varied by varying ρ̅ and γ. Equally spaced values (0.1 rad) of roll, ranging in the interval −1.6 rad ≤ ρ̅ ≤ 1.6 rad with γ = −0.1, −0.3, −0.5, −0.7, −0.9, were used. In degrees, this corresponds to −90° < ρ̅ < 90° and −81° < Ω̅ < 81°. With the above-given parameters, we generated biased potentials for umbrella sampling.^48,49^ The two-dimensional free energy landscape of the cgDNA+ model was computed using the Weighted Histogram Analysis Method, described below, to merge data from different runs. The configurations obtained from biasing cgDNA+ were also used as input for all-atom simulations and transformed into PDB files for all-atom simulations using the x3DNA software.^33,50^ x3DNA maps the rigid base coordinates into all-atom structures.

### All-Atom System Preparation

The standard Molecular Dynamics procedure includes the preliminary steps summarized here. All work is done using the version 2022.5 of GROMACS and version 2.8.3 of PLUMED. The topology file was created using the Amber99 parmbsc1 force field.^51,52^ The DNA strands were placed into a dodecahedral box, leaving 2.0 nm of extra space on both sides of the molecule, with periodic boundary conditions and the water model TIP-3P,^53^ nonbonded interactions are regulated with a cutoff of 1 nm, and for electrostatics, the PME model is used.^54^ The system is solvated in a 150 mM NaCl solution, after which the overall charge was neutralized. In the simulation box, this corresponds to 63 Na^+^ ions and 41 Cl^–^ ions. The following steps were performed using a time step Δ*t* = 2 fs with a Leapfrog integrator and employing LINCS constraints. These steps include energy minimization with a tolerance of 1000 kJ/mol, equilibration in the NVT ensemble for 100 ps while maintaining a temperature of 300 K through a velocity rescaling thermostat, and lastly another 100 ps of equilibration in the NPT ensemble, with the temperature held constant at 300 K, and the pressure fixed at 1.0 bar using a Parrinello–Rahman barostat. In these two short equilibration runs, no bias is applied.

### Biasing All-Atom Simulations: The RBB-NA Algorithm

The Rigid Base Biasing for Nucleic Acids (RBB-NA) algorithm is designed to enhance molecular dynamics (MD) simulations of nucleic acids, such as DNA and RNA.^39^ It allows one to impose specific structural deformations by biasing the rigid base parameters, such as those in eq 4. While biasing cgDNA+ is a relatively simple task, RBB-NA has to turn the bias of eq 4, which is on the coarse-grained coordinates, into the appropriate forces acting on atoms. These biasing forces on atoms need to be imposed at every MD time step. For more details on how this is done, see ref 39. The RBB-NA algorithm is available as a PLUMED package.^55^ Similar biasing schemes were proposed in some earlier works.^56–58^ Here, we run the RBB-NA under the same bias as those imposed on cgDNA+ to obtain the input conformation (same ρ̅ and γ) but with a different stiffness parameter *K*. In the RBB-NA, we used *K* = 100 kJ/mol, which is 10 times smaller. A larger *K* was used in cgDNA+ as this model is considerably stiffer than the actual all-atom MD at high deformations (see Results section); therefore, one needs a stronger constraint to bias cgDNA+. A softer constraint in RBB-NA simulations allows one to sample efficiently a wider range of twist and roll values and to obtain overlapping histograms from umbrella sampling. This, in turn, results in a more accurate calculation of the free energies. Figure 3 shows time traces of roll ρ measured during 1 ns RBB-NA simulations to illustrate the quality of relaxation under these constraints. The simulations are performed after the two 100 ps equilibration runs in the NVT and NPT ensembles described above. On top of each plot of Figure 3, the value of the biased roll ρ̅ is given, while γ = −0.1. The roll measured in the simulation run is, in absolute value, smaller than the biased one, |⟨ρ­(*t*)⟩| < |ρ̅|. This is because the total free energy is given by the contribution of the intrinsic one (as given by eq 3) plus that of the bias, with the former favoring small ρ’s. A positive/negative bias ρ̅ drifts the simulated average roll ⟨ρ­(*t*)⟩ to assume positive/negative values. Most of the runs indicate that the starting point is already equilibrated, although occasionally we observe a drift, as is the case for ρ̅ = −57.3° in Figure 3. Longer runs to 5 ns, however, provide overlapping free energies as those from 1 ns (see Figure S4 of the Supporting Information). In our analysis, we perform multiple 32 runs for each of the five γ, resulting in a total of 0.16 μs per sequence. The simulations with different ρ̅ and γ produce overlapping histograms, which are analyzed using the Weighted Histogram Analysis Method.

**Figure 3:**
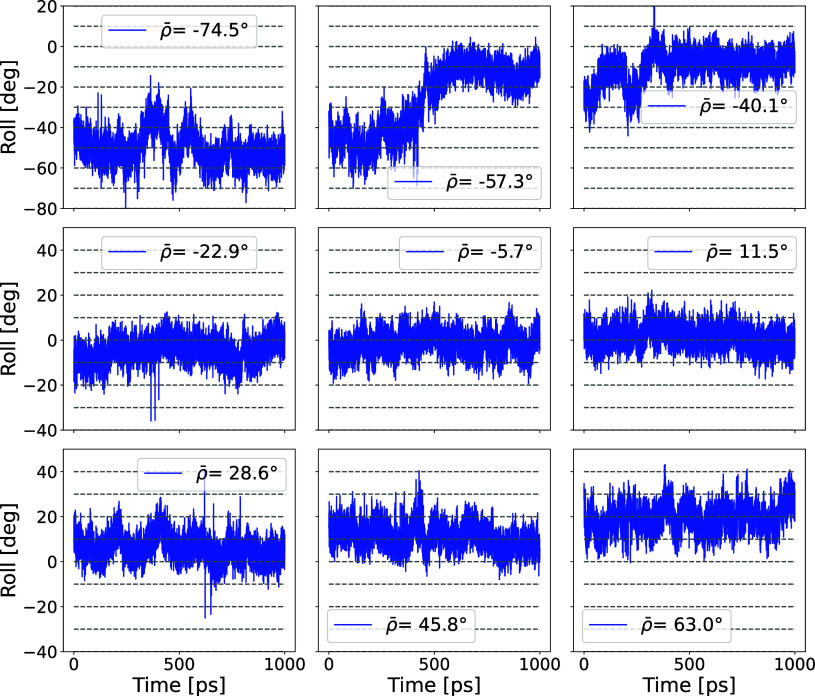
Plots of roll vs time for 1 ns RBB-NA simulation runs for Sequence 1 with different biases ρ̅, with values given at the top of each graph, and fixed γ = −0.1. We recall that the bias in the excess twist is Ω̅ = γρ̅. The roll ρ­(*t*) follows the imposed bias, but note that ⟨|ρ­(*t*)|⟩ < |ρ̅|. In the runs, many overlapping histograms are produced, from which Δ*F*(ρ, Ω) can be calculated. The runs indicate that they are well equilibrated with occasional slow relaxation observed at negative ρ̅, which is discussed in the Results section.

### The Weighted Histogram Analysis Method (WHAM)

The Weighted Histogram Analysis Method (WHAM) is a statistical technique through which one can merge data from multiple umbrella sampling windows to generate an estimate of the free energy profile.^59,60^ In the present work, a two-dimensional free energy landscape is calculated as a function of the roll and twist coordinates. WHAM combines histograms from different biased simulations and iteratively reweights them to reconstruct the unbiased probability distribution *P*(ρ, Ω), from which the free energy is obtained as:

5 $$\Delta F\left(\rho ,\Omega \right)=-k_{B}T\log\frac{P\left(\rho ,\Omega \right)}{P\left(\rho_{\min},\Omega_{\min}\right)}$$

## Results

### cgDNA+ Free Energies

Figure 4 shows a contour plot of the free energy Δ*F*(ρ, Ω), in *k*_B_ *T* units, of Seq 1 from Table 1 as obtained from a Monte Carlo simulation of the coarse-grained cgDNA+ model.^45^ The results are given by (a) an unbiased simulation and (b) biased simulations with a biasing potential given by eq 4. The bias values of ρ̅ are equally spaced (with −91.7° ≲ ρ̅ ≲ 91.7° [1.6 **rad**]) and calculated along five lines of fixed slopes γ = −0.1, −0.3, −0.5, −0.7, and −0.9. The red lines shown in Figure 4(b) correspond to γ = −0.1 and γ = −0.9. In the unbiased case (a), the range of roll and twist sampled by the simulation is limited to |ρ| ≲ 20° and |Ω| ≲ 20°. In the biased case (b), one can obtain a good estimate of Δ*F* for a much wider range of ρ and Ω. As expected, the shape of the free energy is quadratic as cgDNA+ uses quadratic terms to describe interactions between the coarse-grained degrees of freedom. The contour lines of the quadratic model are ellipses with axes that are tilted with respect to the ρ and Ω axes. The “tilted” landscape is due to the coupling between roll and twist discussed in the introduction (the factor *G*_*n*_ in eq 3). The results of the cgDNA+ calculations are useful as a reference for comparison with the all-atom free energies obtained from the RBB-NA algorithm. They are also a good test of the biasing scheme and the performance of WHAM, which is used to combine the results of the different simulations. The WHAM software used for cgDNA+ data analysis is the same as that used in the biased all-atom simulations discussed next.

**Figure 4:**
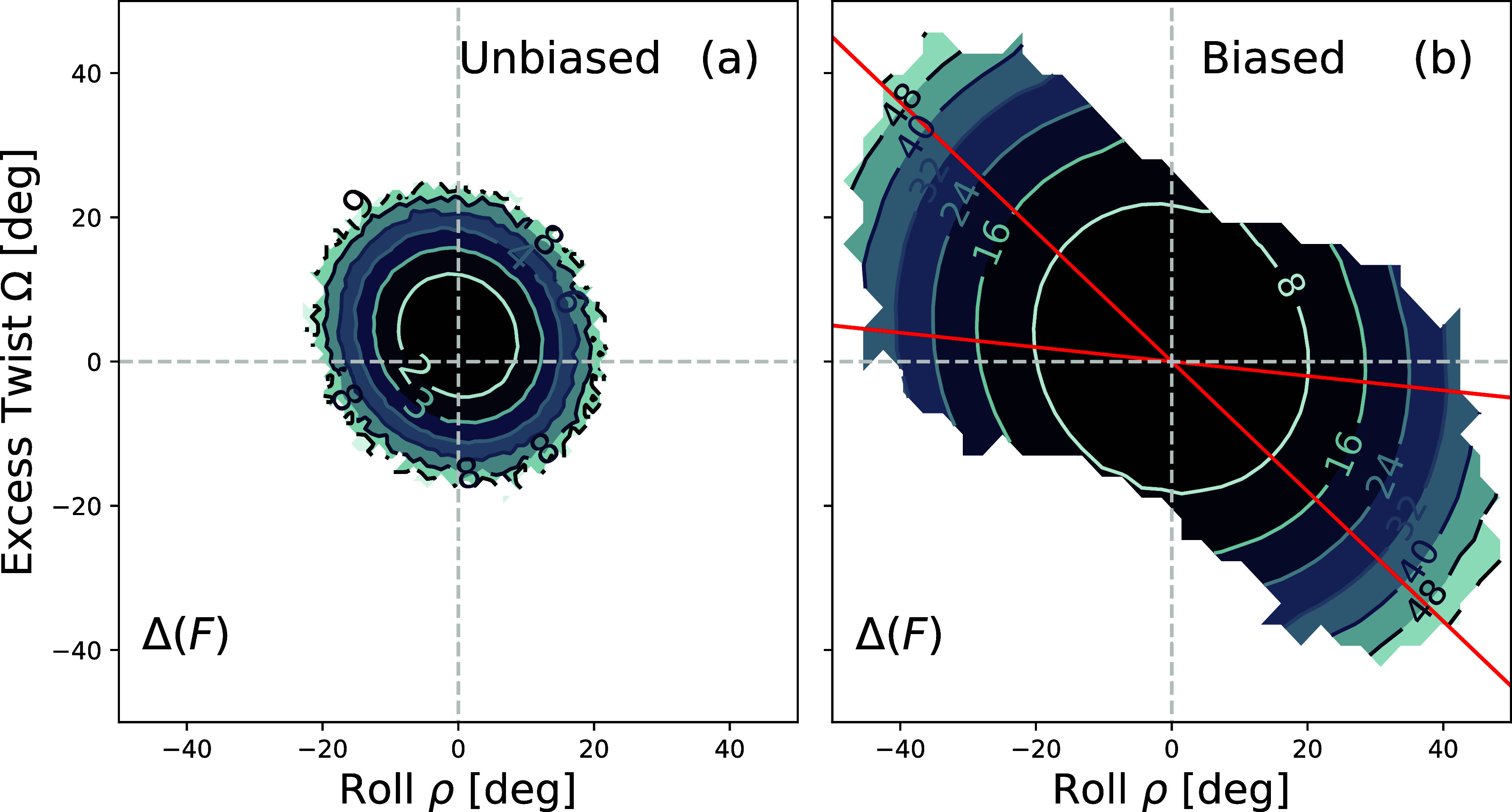
Contour plots of the free energy Δ*F*(ρ, Ω) for the coarse-grained cgDNA+ model for Sequence 1. (a) Unbiased simulation. (b) Biased simulation with a biasing potential given by eq 4 for some fixed ρ̅ and −0.9 ≤ γ ≤ −0.1. The red lines indicate the smallest and largest γ’s. The numbers along the contour lines indicate the free energies in *k*_B_ *T* units. In the unbiased case (a), the free energy extends up to 9 *k*_B_ *T*, while in the biased case (b), the free energy extends up to 56 *k*_B_ *T*.

### All-Atom Model Free Energies from RBB-NA Simulations

We now turn to the results of the all-atom simulations obtained by the RBB-NA algorithm,^39^ as described in Materials and Methods section. Contour plots of the free energies Δ*F*(ρ, Ω) for all six sequences are shown in Figure 5. The data are presented in units of *k*_B_ *T*. All sequences are subject to an identical bias, which is the same as that used to calculate the free energy of the coarse-grained cgDNA+ model of Figure 4. The only difference is that a smaller *K* in eq 4 is used in the RBB-NA algorithm to allow sampling of a more extended region in the ρ–Ω plane; see Materials and Methods section.

**Figure 5:**
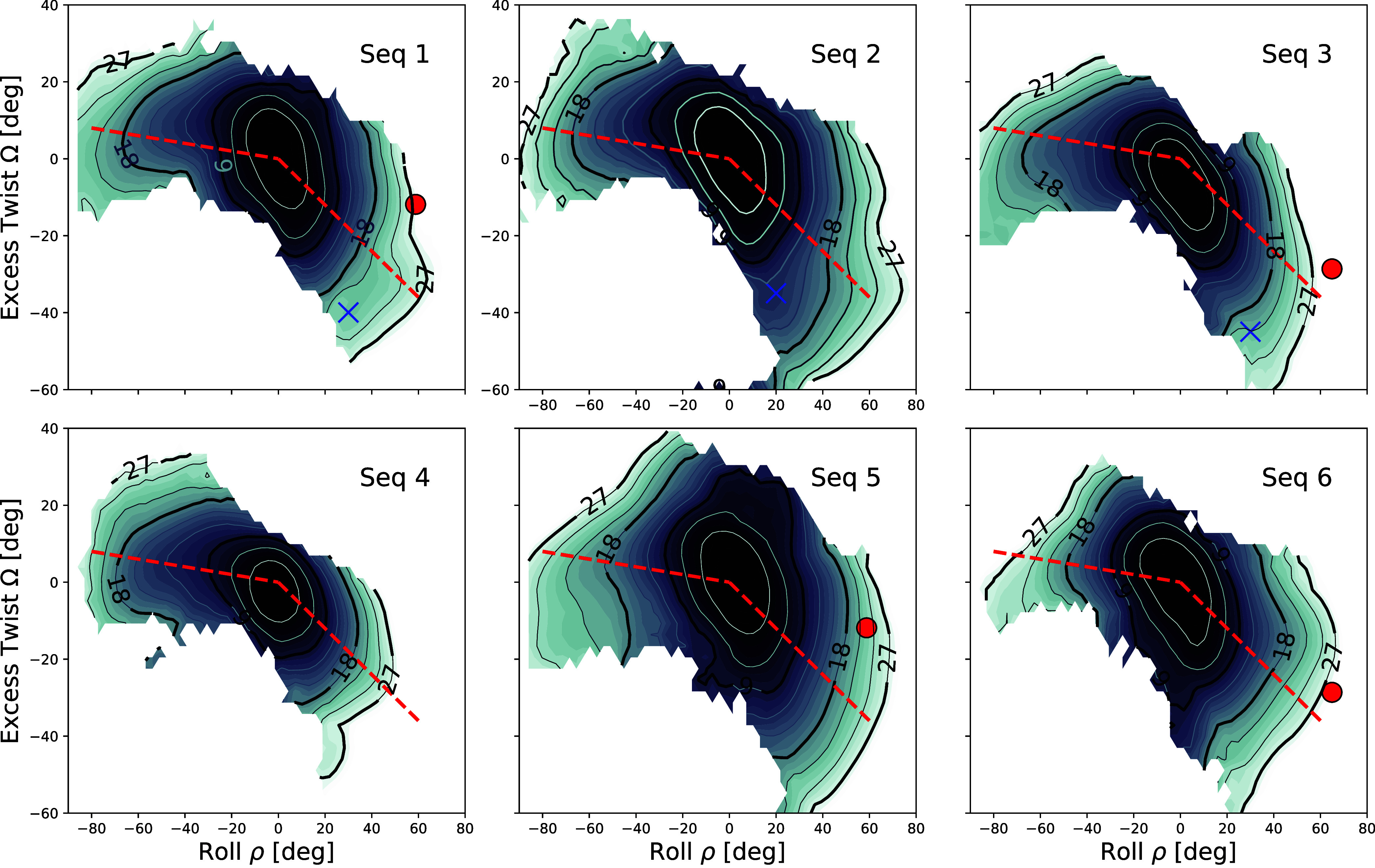
Contour plots of the free energy landscapes for the six sequences studied, obtained by the RBB-NA algorithm via umbrella sampling and WHAM analysis. The free energies are given in units of *k*_B_ *T*, and the contour lines correspond to equal free energy levels, shown every 3 *k*_B_ *T* and up to 27 *k*_B_ *T*. The numbers printed above the lines give the corresponding free energy values. In sequences 1 and 3, the red symbols indicate the values of roll and excess twist derived from the protein-bound DNA crystal structure ρ = 59°, Ω = −12° for Seq 1 (IHF, PDB: 1IHF) and ρ = 65°, Ω = −29° for Seq 3) (Hbb, PDB: 2NP2). The blue crosses indicate regions of the landscape that tend to flatten, which we flagged as potential kinks. The values of roll and twist for these are given in Table 2. The red dashed lines mark the range of inclinations over which the free energy profile is depicted in 1D in Figure 6.

#### General Features

We first discuss some common features of the free energies shared by all sequences analyzed. For small roll and small excess twist, the RBB-NA free energies for all sequences analyzed approximately follow the quadratic model behavior of the coarse-grained cgDNA+ model of Figure 4(b). In RBB-NA for small deformation parameters, the landscapes appear to be tilted with respect to the ρ and Ω axes due to the effect of a positive twist–roll coupling. At larger deformations, the RBB-NA free energy landscapes become asymmetric under the change ρ → −ρ, Ω → −Ω (unlike the cgDNA+ model, which uses quadratic free energies and is therefore symmetric by construction, see Figure 4). This behavior is shared by all sequences: for positive roll, the landscape remains “tilted”, but for negative roll, free energies tend to become symmetric around the *y*-axis (twist). The dashed red lines are Ω = −0.1 ρ (ρ < 0) and Ω = −0.6 ρ (ρ > 0) and are drawn as a guide to the eye to indicate the degree of asymmetry between the positive and negative ρ. In the regime of strong deformation, the quadratic approximation (eq 3) breaks down, and higher terms are needed to describe the free energy (see Discussion section). While the quadratic free energy of eq 3 is invariant under a roll and twist change of sign ρ → −ρ, Ω → −Ω, cubic terms such as Ω^3^, ρΩ^2^··· will break this symmetry. We recall that the bias is applied along lines of a constant negative slope in the ρ–Ω plane (see Figure 4(b)). This bias is applied in a symmetric range of values: −ρ̅_max_ ≤ ρ̅ ≤ ρ̅_max_ and −Ω̅_max_ ≤ Ω̅ ≤ Ω̅_max_. Forcing the system into extreme deformations with high twist and roll can disrupt base pairing in the DNA double helix in an RBB-NA simulation. These broken conformations are discussed in the Supporting Information and are excluded from the calculation of the free energy landscapes. We note that despite the symmetry in the bias, this generates an excess twist below −40°, but hardly above 20°, showing that overtwisting DNA becomes energetically more costly than undertwisting in the high deformation regime.

#### Sequence-Dependent Effects

We now turn to discussing the sequence-specific properties of the free energies. For Sequences 1 and 3, we showed, as red symbols in Figure 5, the values of twist and roll measured at the kinks in the crystal structure of the sequences when bound to IHF (Seq 1) and Hbb (Seq 3), respectively. As mentioned earlier, because of the nick present in the crystal structure, these data are not available for Seq 2 (see Figure 1). Although our main interest is in bare DNA properties at large |ρ| and |Ω|, it is useful to consider kinks in protein–DNA complexes. In these complexes, kinks are characterized by large positive roll and negative excess twist, corresponding to undertwisting.^34^ For a more quantitative comparison between the different sequences, we have plotted in Figure 6 the free energies along the two red dashed lines in the ρ–Ω plane of Figure 5. Curiously, Sequence 4 (DDD) has the highest free energy of all other sequences for ρ > 0 and the lowest of all for ρ < 0. There is, in general, less sequence variability for ρ < 0 than for ρ > 0. We see no signatures of local minima in our free energies, but there are, in several sequences, inflection points in which the free energy landscape tends to flatten. This is close to the behavior of the hinge model (eq 2), which is built on two separate free energy branches. The only two sequences with inflections in the free energy for ρ > 0 are those that show kinks at positive roll when bound to IHF (Sequences 1 and 2). To facilitate a comparison of roll and twist between crystallographic structures and RBB-NA simulations, we compile the relevant values in Table 2, which lists data for Sequences 1, 2, and 3 from the kinked IHF and Hbb structures. In the simulations, tentative kinks are identified as regions in which the two-dimensional landscape tends to flatten. We indicate these points as blue crosses in Figure 5. Overall, kinks in protein–DNA complexes tend to exhibit larger roll angles and are less undertwisted than those identified as kinks in the RBB-NA simulations. Protein binding is expected to perturb bare DNA behavior.

**Figure 6:**
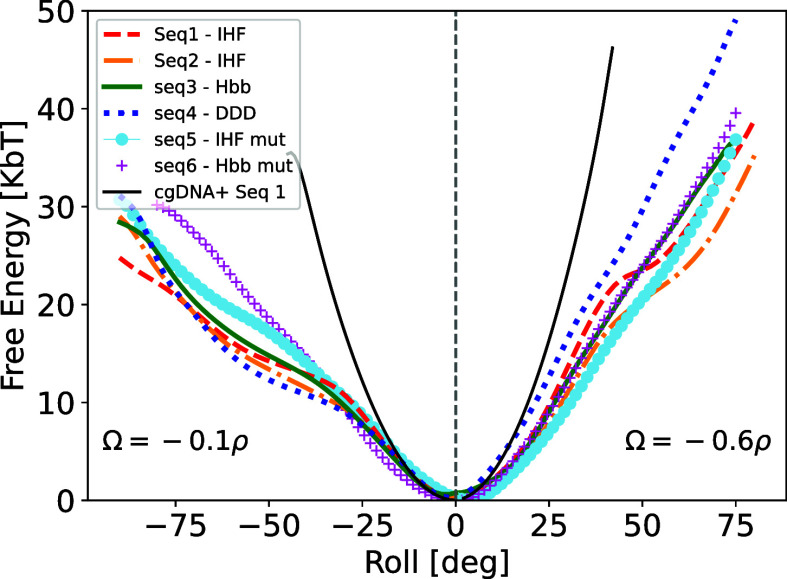
Free energies of the six sequences calculated with the RBB-NA algorithm plotted along the red dashed lines shown in Figure 5 with slopes Ω = −0.1ρ (shown for ρ < 0) and Ω = −0.6ρ (shown for ρ > 0). For a description of individual behavior, see the text. The thin solid black line is the (quadratic) free energy from the cgDNA+ model for Seq 1. This model overestimates the actual all-atom free energy data, showing that the DNA is much more deformable than predicted by the harmonic approximation.

**Table 2:** Comparison of Roll/Twist Parameters for Kinks as Obtained from Biased RBB-NA Simulations vs Crystallographic Data from Protein/DNA Interactions^a^

	Simulations			Cryst. Struct.	
	Δ*F*(*k*_B_ *T*)	roll	twist	roll	twist
Seq 1	21	30	–5	60	20
Seq 2	13	20	0	-	-
Seq 3	22	30	–10	40	20

a The kinks in simulation are identified as regions where the landscape tends to flatten and are shown as blue crosses in Figure 5. It is worth noting that the value for the reaching roll of ∼60° has been estimated from all-atom MD^56^ to be ≃25*k*_B_ *T*. Other authors have estimated that this value should be close to ≃15*k*_B_ *T*, based on the observation that kinks compete with base-pair disruption to alleviate strain.^22^

In all sequences analyzed, we found that cgDNA+ free energies are always larger than the corresponding RBB-NA free energies. In addition, as mentioned earlier, cgDNA+ free energies are quadratic in ρ, while, apart from local “flattening” behavior at some angles, all-atom data tend to increase linearly with the bending angle. This was observed in earlier simulations as well^39,56^ and is reminiscent of the linear subelastic chain behavior,^6^ which uses a free energy *g*(θ) = α|θ| in eq 1.

### Beyond the Harmonic Approximation

The free energy data from Figures 5 and 6 indicate that the quadratic model of eq 3 breaks down for roll and twist angles |ρ|, |Ω| ≳ 10°. To explore the nature of the anharmonic effects, we analyzed the free energies using higher order powers in ρ and Ω via the following model:

6 $$\beta \varepsilon_{n}=\frac{1}{2}\left(A_{2}^{\rho }\rho^{2}+C_{2}\Omega^{2}+2G\Omega \rho +A_{3}^{\rho }\rho^{3}+C_{3}\Omega^{3}+B\rho^{2}\Omega +D\rho \Omega^{2}+A_{4}^{\rho }\rho^{4}+C_{4}\Omega^{4}+H\rho^{2}\Omega^{2}\right)$$

where we omit for simplicity the index *n* from ρ, Ω and from the stiffness parameters $A_{2}^{\rho }$ , *C*_2_, *G*, $A_{3}^{\rho }$ , *C*_3_··· While neglecting stiff tilt degrees of freedom, the model (eq 6) generalizes (eq 3) by including all possible cubic terms obtained by combining ρ and Ω. Quartic terms were also considered, leaving out ρ^3^Ω and ρΩ^3^ as these were not found to significantly improve the fit. Figure 7 shows the contour plot of the free energy landscape for Seq 4 for Δ*F* ≤ 9*k*_B_ *T* (solid lines) and the fitted data for Δ*F* ≤ 7*k*_B_ *T* (dotted lines). The harmonic model fits well with free energies in the range ≲3*k*_B_ *T,* and we used data in this range to estimate the three quadratic parameters $A_{2}^{\rho }$ , *C*_2_, and *G*. Keeping these parameters fixed, we then fitted both a cubic model (see Supporting Information) and the full model described by eq 6. As seen in Figure 7 (right), the anharmonic model captures well the all-atom (RBB-NA) free energies and also the anisotropies between the positive and negative roll regions. A more extended analysis of fits and their quality from quadratic, cubic, and quartic models for other sequences is given in Supporting Information.

**Figure 7:**
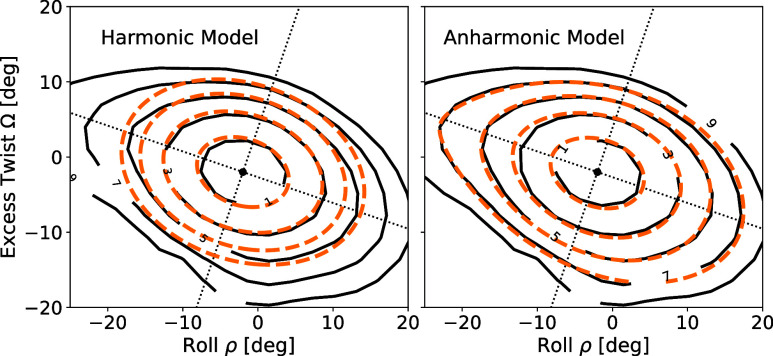
Solid black lines: Contour plots of the RBB-NA free energies for Seq 4 (DDD). Dotted orange lines: Fitted values for the harmonic model (left) in the region Δ*F* ≤ 3*k*_B_ *T* and for the anharmonic model (right) in the region Δ*F* ≤ 7*k*_B_ *T*. The dotted lines are the principal axes of the ellipses, which are contour lines of the harmonic model.

One of the remarkable features of the coefficients in Table 3 is $C_{3}\gg A_{3}^{\rho }$ , indicating the presence of a strong cubic term in the twist (∼Ω^3^) as opposed to a very weak cubic roll term (∼ρ^3^). The same holds for all other sequences (see Supporting Information). The positive *C*_3_ suppresses overtwisting (Ω > 0) and favors undertwisting (Ω < 0) of the molecule. A large asymmetric term in twist is perhaps not surprising in view of the chirality of the DNA molecule, which is a right-handed helix in its B-form. Such a contribution is expected from stacking interaction, as undertwisting increases stacking.

**Table 3:** Coefficients Obtained from Fitting the Anharmonic Model (eq 6) to the Free Energies of Seq 4 in the Range Δ*F* ≤ 7*k*_B_ *T* in Successive Steps Starting from a Quadratic Model, as Explained in the text^a^

	$A_{k}^{\rho }$	*C*_*k*_	*G*	*B*	*D*	*H*
quadratic (*k* = 2)	5.6	9.7	1.6	-	-	-
cubic (*k* = 3)	0.2	10.5	-	–2.5	–1.6	-
quartic (*k* = 4)	–4.3	–4.3	-	-	-	–8.7

a Note: quadratic coefficients are multiplied by a factor 10^–2^, cubic coefficients by a factor 10^–4^ and quartic coefficients by a factor 10^–5^. The data are in dimensionless units, as they are obtained by fitting angles in degrees.

In our expansion, quartic terms are negative, (see Table 3) which lower the free energies further for high deformations. Usually, in field theory descriptions, the highest-order terms must have positive prefactors, which ensure that ε_*n*_ → +∞ for large |ρ| and |Ω|. This stability condition is not needed in this case, as the free energy (eq 6) describes a free energy manifold of unbroken conformations, which only exists for a finite range of |ρ| and |Ω|.

## Discussion

In this paper, we analyzed extremely bent DNA via all-atom MD simulations. Such highly deformed conformations cannot be observed in unbiased simulations of linear DNA within the currently reachable time scales. Prior work considered simulations of DNA minicircles of 60–100 bp.^16,17^ These molecules are already bent in their initial conformation, and this bending stress facilitates the formation of sharp kinks within relatively short time scales (∼80 ns). An alternative approach was to bend a linear DNA molecule by imposing restraints at its two ends.^19^ The induced bending in the latter biasing scheme is distributed along the sequence over a few base pairs, and the determined free energy is associated with a global bending angle rather than a localized deformation. Here, we applied a recently developed algorithm, the RBB-NA,^39^ which allows one to bias any of the 12 rigid base coordinates in all-atom simulations. Although it is possible to bias multiple sites simultaneously, we restricted our analysis here to the local biasing of the roll and twist coordinates of the central nucleotide pairs of six dodecamers.

### Asymmetric Free Energy Landscapes

Figure 8 shows a schematic contour plot of the typical free energy landscape in the roll–twist plane, as obtained from biased MD simulations. At small ρ and Ω, the landscape follows a quadratic model behavior, which is represented by the red ellipse with tilted main axes, as expected from a positive twist–roll coupling (*G* > 0 in eq 3). One of the most striking features is the asymmetric behavior in the positive/negative roll. At positive and large ρ, the landscape remains “tilted” as is the case at small ρ and Ω due to twist–roll coupling. At negative and large ρ the tilting disappears, and the landscape “aligns” along the horizontal (roll) axis, implying that bending with ρ < 0 does not come with a significant twist. Our results also indicate that free energies are smaller in the negative roll region, as opposed to the ρ > 0 region (see Figure 6).

**Figure 8:**
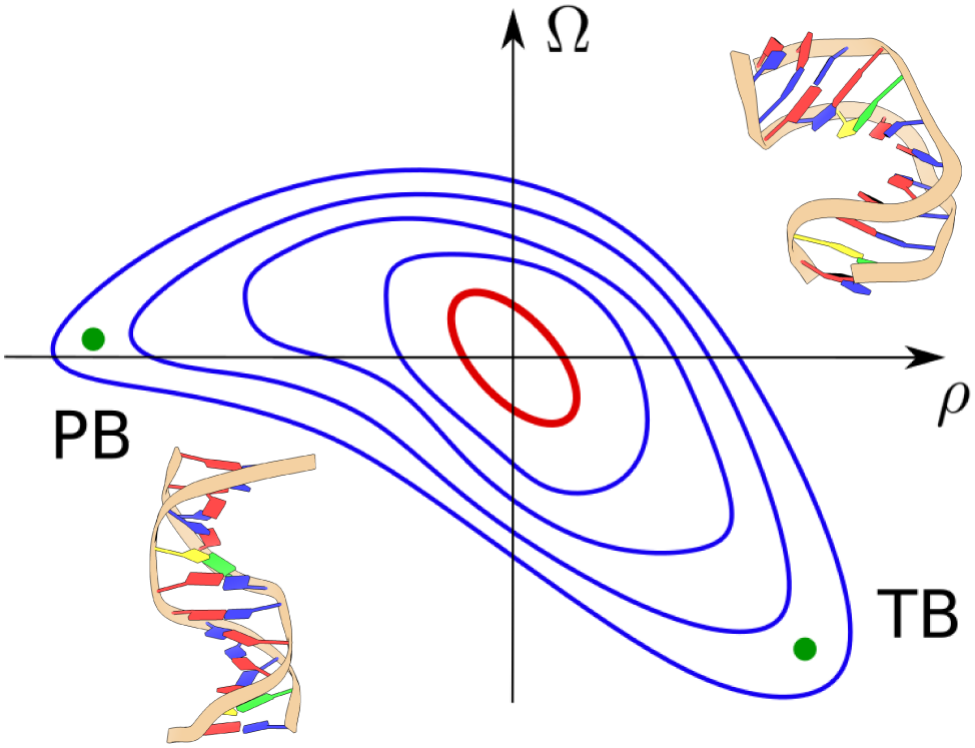
Schematic view of the average behavior of the free energy landscape showing the harmonic regime (red ellipse) and the anharmonic regime (blue curves). Extremely bent deformations with high |ρ| can be obtained by undertwisting at ρ > 0 and without significant excess twist at ρ < 0. We refer to the former as twist-bend (TB) kink and to the latter as pure-bend (PB) kink. The two configurations at the top right and bottom left of the figure are generated using Seq 1 and represent typical structures expected for TB and PB kinks. PB is obtained by imposing a bias ρ̅ = −86° and Ω̅ = −0.1ρ̅. For TB, ρ̅ = 86° and Ω̅ = −0.9ρ̅.

### Twist-Bend Kinks vs Pure-Bend Kinks

The landscape asymmetry suggests that there are two distinct pathways to generate sharp bending (roll) angles. The lowest free energy states for negative and large roll angles correspond to modest associated twist. We refer to these conformations as pure-bend kinks (PB in Figure 8). Conversely, the free energy landscape implies that a large positive roll should have an associated negative excess twist (undertwisting). We refer to these as twist-bend kinks (TB in Figure 8). The latter conformations are commonly observed in DNA–protein complexes,^34^ as in the example of IHF, see Figure 1 where high positive roll is accompanied by undertwisting. While Figure 1 shows a modest undertwisting, we note that the degree of undertwisting can also be higher than that observed for the IHF (see Figures S1 and S2 in Supporting Information). Crystal structures of protein–DNA complexes consist of DNA fragments of ∼20 nucleotides, which are torsionally unconstrained and therefore could accommodate excess twist. Undertwisting is quite common in vivo, as the DNA of most organisms is negatively supercoiled; therefore, it is natural to expect kinks of twist-bend type in protein–DNA complexes in living cells. The other types of kinks (which we referred to as pure-bend kinks), corresponding to large negative roll and modest or no excess twist, were actually observed in simulations of DNA minicircles.^16,17^ Figure S3 in the Supporting Information shows the rotational parameters of a DNA minicircle (data from ref 16) with such a strong negative roll, accompanied by a modest undertwist. We expect that this type of kink would show up in strongly torsionally constrained DNA, as in minicircles.

### Strong Roll/Twist Bias Induces Base-Pair Breakage

When the bias in the RBB-NA algorithm in the roll and twist exceeds some (sequence-dependent) threshold value, the molecule base pairs of the central dinucleotide steps get disrupted (see Figure S8). However, there is a large domain of ρ and Ω values for which the base pairing remains intact, which comprises large bending (roll) angles. Indeed, the free energy landscapes of Figure 5 correspond to conformations in which the base pairs are undisrupted. The simulation data for biases that lead to base pair disruptions are not included in the free energy calculations. The kinks hypothesized by Crick and Klug^1^ and also observed in DNA simulations with minicircles^16,17^ correspond to localized, highly bent structures with intact base pairing. The latter simulations also generated disrupted base pair conformations involving several bp (typically two or three of them), while undisrupted kinks are localized at a single base-pair step.^16,17^

### DNA Is More Bendable Than the Harmonic Model Predicts

In general, we find that the free energy landscapes obtained from all-atom data are substantially lower than those extrapolated from the harmonic model (Figure 6), indicating that DNA is much more deformable than what was predicted from the quadratic approximation. The all-atom landscapes show several inflection points, which depend on the sequences analyzed and are reminiscent of the phenomenological hinge model (eq 2), although we do not observe local ″metastable″ minima in Δ*F* as hypothesized in that model. For roll and twist in the range |ρ|, |Ω| ≤ 20°, the free energy landscapes can be fitted by a higher order model in which cubic and quartic terms in ρ and Ω are included (see eq 6 and Supporting Information). The analysis shows the presence of a strong ∼ Ω^3^ term favoring undertwisting, dominating over a much weaker ∼ ρ^3^ contribution.

### Parametrizing Coarse-Grained DNA Models

Coarse-grained DNA models are widely used because they can reach much longer time scales and sequence lengths than all-atom simulations. Rigid base models such as cgDNA^43^ or analogous models^61,62^ use quadratic interaction energies or multimodal approximations^63,64^ and thus are expected to describe accurately DNA conformations in which the bending deformations are weak. Our analysis provides insights into the free energy landscape properties for a wide range of twist and roll values, which could be used to parametrize rigid base models to go beyond quadratic approximations. Additionally, it would be interesting to probe the elastic response to extreme bending deformations of other coarse-grained particle-based models^65–68^ and to compare them with the all-atom data reported here.

### Probing Force Fields in the High Deformation Regime

Our simulations use a state-of-the-art AMBER parmbsc1 force field,^52^ which, like other DNA force fields, was parametrized for structures close to an ideal B-DNA. A systematic comparison of commonly used atomistic force fields unveiled some differences in predicting elastic properties.^69^ It has been pointed out that one of the shortcomings of the current force fields is the tendency to overstabilize certain interaction terms in the double helix.^70^ In particular, the base-pair stacking interactions were found to be overstabilized relative to experimental measurements.^71^ Since the kinks discussed here involve local base-pair unstacking, an excessively strong stacking term would naturally hinder this process. Consequently, such an overstabilization is likely to lead to an overestimate of the free energy cost of kinking. It is also believed that improved force fields will lead to a higher degree of conformational flexibility.^70^ It would be interesting to test deformation free energies, such as those reported in Figure 5 using alternative force fields. An assessment of recent AMBER-derived DNA force fields (OL21 and Tumuc1) showed that both provide notable improvements over the previous generation.^72^ The RBB-NA algorithm^39^ could handle these as well, and it would be interesting to explore how the free-energy landscapes and DNA kinkability vary across a broader range of force fields.

## Conclusion

In conclusion, we investigated the behavior of highly bent DNA using the RBB-NA algorithm, through which we applied a localized bias potential to the central dinucleotide step of some selected dodecamers. The core contribution of this work lies in the detailed characterization of the free energy landscapes. All analyzed sequences present asymmetric behavior: DNA accommodates undertwisting through a combination of positive roll, whereas overtwisting, particularly when coupled with negative roll, results in significantly higher energetic costs, often leading to base-pair breakage. These results highlight the need for incorporating anharmonic models in DNA structural descriptions, such as the one we presented here. Furthermore, our analysis offers a pathway toward improved parametrization of coarse-grained models.

## Supplementary Material



## Data Availability

The data that support the findings of this study are available at 10.5281/zenodo.17184865. These data include: PDB files generated using cgDNA+, Scripts for running RBB-NA molecular dynamics simulations, and Python scripts for WHAM analysis and figure generation.

## Notes

Additional kink configurations from protein–DNA crystal structures and from DNA minicircle simulations, MD time trajectories, error analysis, details on the fitting procedure, relaxation times, and base-pair disruption analysis (PDF)
