# Supplementary material for: Inferring DNA Kinkability from Biased MD Simulations
Source: J Chem Theory Comput. 2026 Jan 13;22(2):981–92. doi: 10.1021/acs.jctc.5c01660 (PMC12854757; doi:10.1021/acs.jctc.5c01660)
Supplement: Supplementary file 1 [file ct5c01660_si_001.pdf]

Supporting Information: Inferring DNA kinkability from biased MD simulations

Arianna Fassino,*,† Enrico Carlon,*,† and Aderik Voorspoels*,†,‡

†*Soft Matter and Biophysics, KU Leuven, Celestijnenlaan 200D, B-3001 Belgium*

‡*Institute of Systems, Molecular and Integrative Biology, University of Liverpool, Liverpool L69 7ZB, United Kingdom*

E-mail: arianna.fassino@kuleuven.be; enrico.carlon@kuleuven.be; aderik.voorspoels@liverpool.ac.uk

1 Kinks in protein-DNA complexes from crystal structures

It is known, as it was pointed out in early analysis of crystal structure data [**Olson 1998**], that sharp kinks in DNA-protein complexes tend to have positive roll and negative twist. Figures S1 and S2 show two additional examples (besides the IHF shown in the main text) of crystal structures of DNA complexes having each two sharp kinks for the Hbb-DNA and CAP-DNA complexes, respectively. These data show similar features as those observed in IHF: the kinks in protein-DNA structures are indeed characterized by a large positive roll and a negative excess twist. Both kinks are localized at a single basepair step, shown in grey in Fig. S1 and S2(b). The CAP-DNA complex is characterized by a smaller roll angle at

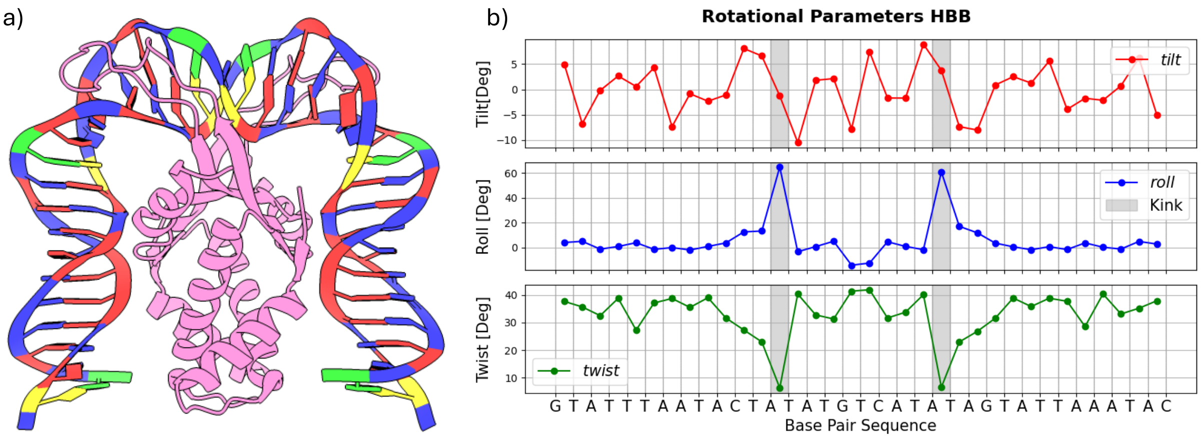

Figure S1: (a) Histone-like protein from Borrelia burgdorferi (Hbb in short) is a DNA binding-protein. The figure shown the three dimensional crystallographic structure of Hbb bound to DNA as from Protein Data Bank [**Mouw and Rice (2007)**]. (b) Plots of Rotational coordinate tilt(τ), twist, and roll (ρ) as obtained using W3DNA. The DNA has two sharp kinks, localized in two symmetric AT step (grey area). As for IHF (see Fig. 1) main text, the kinks are characterized by a large positive roll ($\rho \approx 60°$) and a negative excess twist $\Omega \approx -20°$. Tilt varies very weakly along the sequence, with typically $|\tau| \lesssim 5°$.

the kinks $\rho \approx 40°$ as opposed to the Hbb-DNA $\rho \approx 60°$. In both complexes tilt varies very weakly $|\tau| < 10°$, as opposed to the strong variation in roll and twist.

DNA kinks were also discussed in the context of nucleosomes. It was found that, particularly for some sequences, the DNA contains some sharp bends at specific sites. Figure S3 shows roll and twist for two nucleosomal DNA from crystallographic data of the the Widom 601 strong positioning sequence [**Vasudevan et al 2010**]. These show two different fragments of the same sequence, for two different crystal orientations. Base pair steps with large roll are indicated in grey. At kinks location the roll is negative in these examples. The corresponding twist is higher than average in five cases (as expected from twist-bend coupling), while it is not significantly perturbed in two cases. While we show these examples here for completeness, we emphasize that the DNA substrate in the nucleosome is overall bent, which may lead to some differences to the localized sharp bents discussed in this work, in which the bias is exclusively applied to one specific base-pair step at the center of a dodecamer.

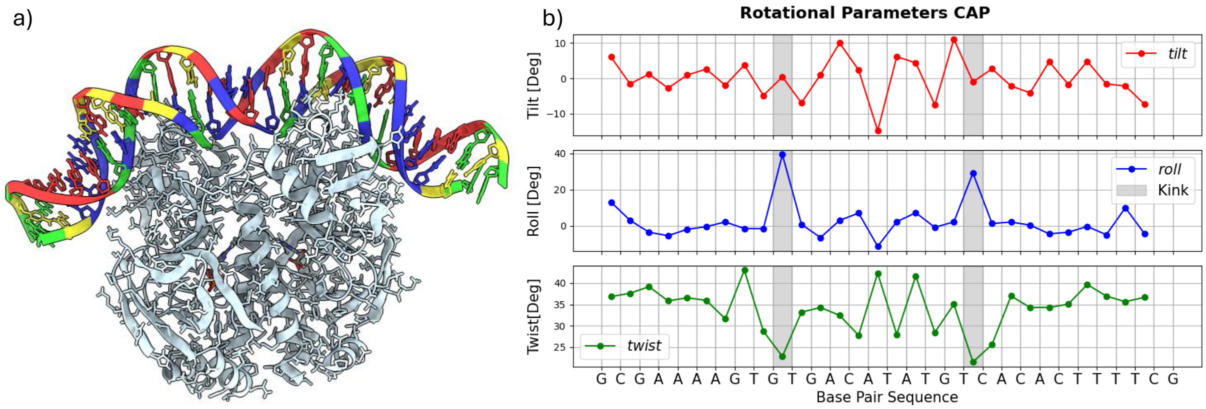

Figure S2: Catabolite Activator Protein (CAP) is a bacterial transcriptional activator in E. Coli and other bacteria [**Schults (1991)**]. (a) Three dimensional crystal structure of CAP-DNA. (b) Plots of Rotational coordinate tilt(τ), twist, and roll (ρ) as obtained using W3DNA. At the two kinks these show similar features as for the IHF (Fig. 1 main text) and Hbb (Fig. S1): positive roll $\rho \approx 40°$, although not as large as the IHF and Hbb, and negative excess twist.

2 Kinks in DNA minicircles

Simulations of DNA minicircles reported different types of kinks structures [**Lankas et al 2006**, **Mitchell et al 2011**]. Kinks with undisrupted base pairs were reported to have predominantly negative roll. Figure S4(a) shows a snapshot of the configuration of a minicircle of 120 base pairs after 80 ns of MD simulation. A plot (b) of the rotational parameters tilt, roll and twist shows the presence of a sharp kink with large negative roll ($\rho \approx -90°$) and small negative excess twist. This conformation supports the report of asymmetric free energy of DNA deformations of Fig. 5 of the main paper: undertwisting is favored to overtwisting.

3 RBB-NA biased runs

The Figure S5 shows some additional plots of roll (ρ) [deg] vs. time [ps] for various sequences and gammas. On top of each graph we printed the corresponding values of the bias applied, $\bar{\rho}$. Compared to Fig. 3 of the main text here we included different sequences combined with higher γ, implying a stronger bias on the twist as $\bar{\Omega} = \gamma\bar{\rho}$. As discussed in the main text,

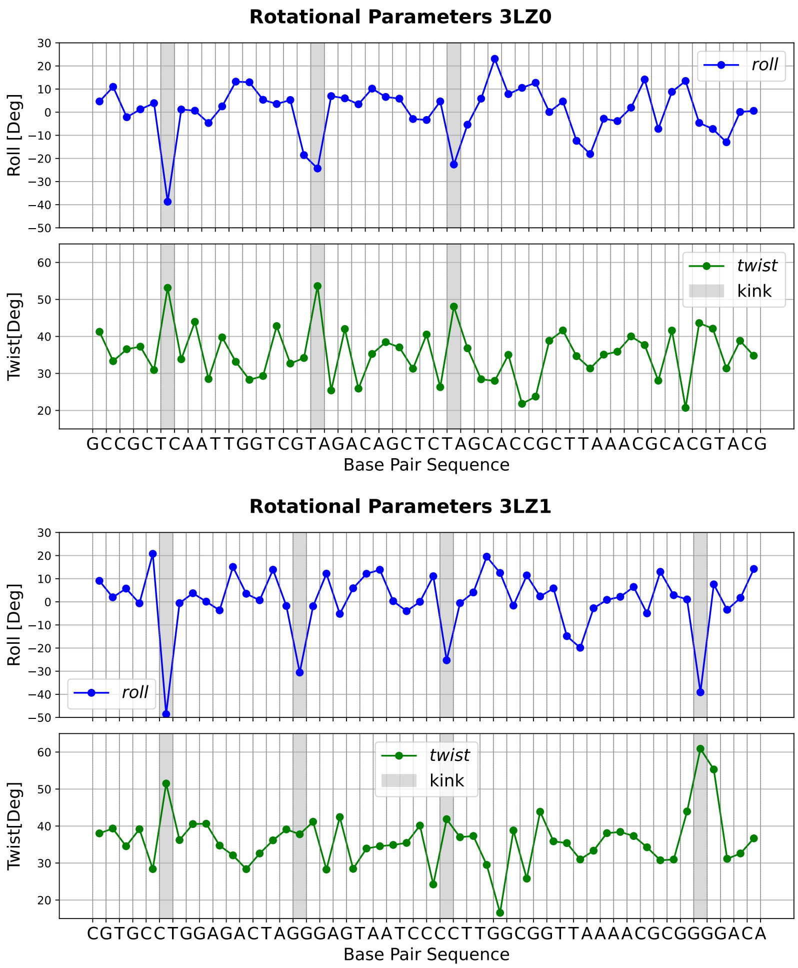

Figure S3: Rotational Parameters Twist and Roll for nucleosomal DNA from pdb entries 3LZ0 and 3LZ1. The sequences are from the 601 Widom positionin sequence.

$\rho(t)$ fluctuates and follows the applied bias $\bar{\rho}$, but as previously evidenced $\langle|\rho(t)|\rangle < |\bar{\rho}|$. Apart from some fluctuations, the system seems to be well equilibrated after the NVT and NPT prepararion runs described in the Methodology Section of the main text. As seen also in Fig. 3 of the main, $\rho(t)$ has stronger fluctuations for some specific values of the bias.

4 Sampling free energies from longer simulation runs

RBB-NA simulation time is fixed at 1 ns for any values of applied bias $\bar{\rho}$, $\bar{\Omega} = \gamma\bar{\rho}$. To test whether such time interval is sufficient to probe equilibrium free energies, we repeated the calculations for a 5 ns simulations for Sequence 2 of Table 1. Figure S6 shows a plot of roll vs. time for two simulations of 1 ns (left) and 5 ns sampling for Seq. 1. The left

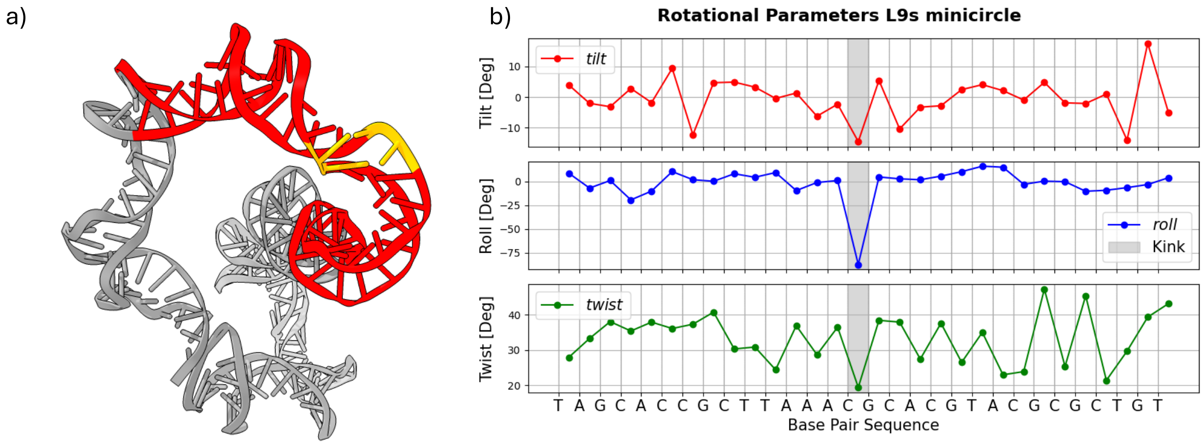

Figure S4: a) Three dimensional structure of Minicircle L9s as presented in the Supplementary Data in [**Lankaš et al. 2006**]. In red we show the base pairs in the vicinity of a sharp kink (yellow) for which the rotational parameters were computed. b) Plots of Rotational coordinate tilt(τ), twist, and roll (ρ) as obtained from the structure in a) using W3DNA. The type I kink is in the correspondence of CG step. We observe a negative roll ($\rho \simeq -87°$) and undertwist, corresponding to a negative excess twist ($\Omega \simeq -14°$).

figure is the same as Fig. 3 in the main text. Figure S7 (top row) compares the free energy landscapes as obtained for 1 ns (left), 5 ns (center) and the last 4 ns of the 5 ns simulation (right). For a more quantitative comparison we plotted the free energies along three lines (Fig. S6, bottom row) with positive roll and negative excessive twist defined by $\rho = -0.2\,\Omega$, $\rho = -0.5\,\Omega$ and $\rho = -0.8\,\Omega$. The three datasets show good overlap with some differences at high roll ($\rho \approx 50°$) along the direction $\rho = -0.5\,\Omega$. The longer sampling time free energies shows a more pronounced "flattening" while the 1 ns as a gentler inflection point.

5 Boostrap Analysis

We discussed in the Method Section of the main text the umbrella sampling approach. Simulations of 1 ns were run for 160 different biases $\bar{\rho}$ and $\bar{\Omega}$. They were later combined using WHAM analysis to get the free energy landscape. We perform here a boostrap analysis to get insight on the statistical convergence of the WHAM. Bootstrapping is a resampling statistical technique to estimate confidence intervals using the empirical distribution of data. It involves generating dataset by sampling with replacement from the original simulations

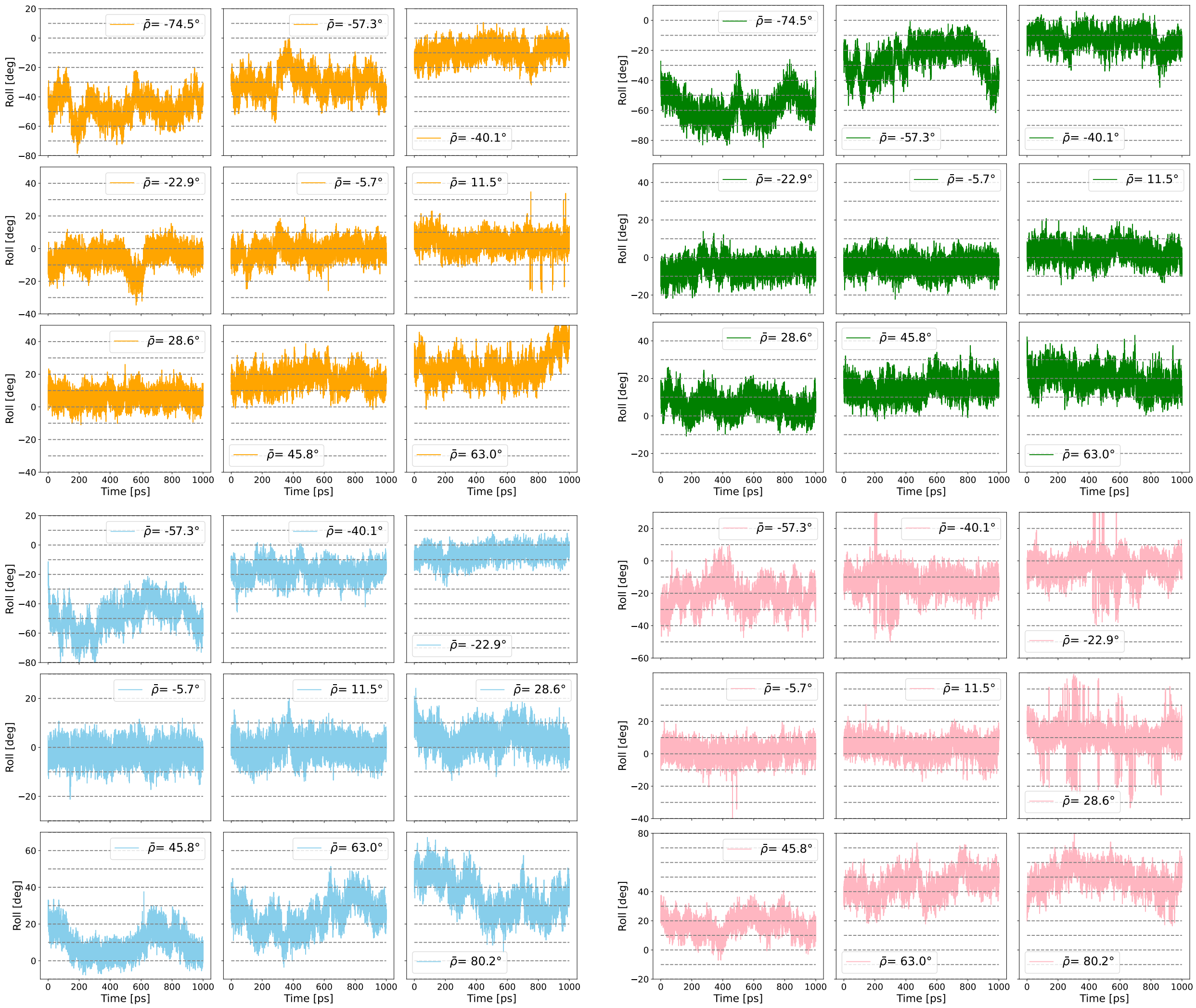

Figure S5: Examples of relaxation of the systems for 4 different sequences and gammas. Color code: Orange represents sequence 2 with $\gamma = -0.3$, Green is sequence 3 and $\gamma = -0.5$, Sky blue sequence 4 $\gamma = -0.7$, Pink sequence 5 $\gamma = -0.9$.

data. Standard bootstrapping assumes individual observation to be independent. Here the data are correlated as they are generated from MD trajectories, so we employed the block bootstrapping approach. Indeed, we resampled within each umbrella window separately. For every window, we generated 100 bootstrap replicas by resampling ρ and Ω values with replacement. For each replica, we reconstructed the WHAM calculation to get the free energy landscape. The resulting ensemble was then used to compute the mean Free Energy value, for each point of the grid, and the 95% confidence interval (CI).

Figure S8 presents the results of the bootstrapping analysis for sequences 1, 2, 3 and 4.

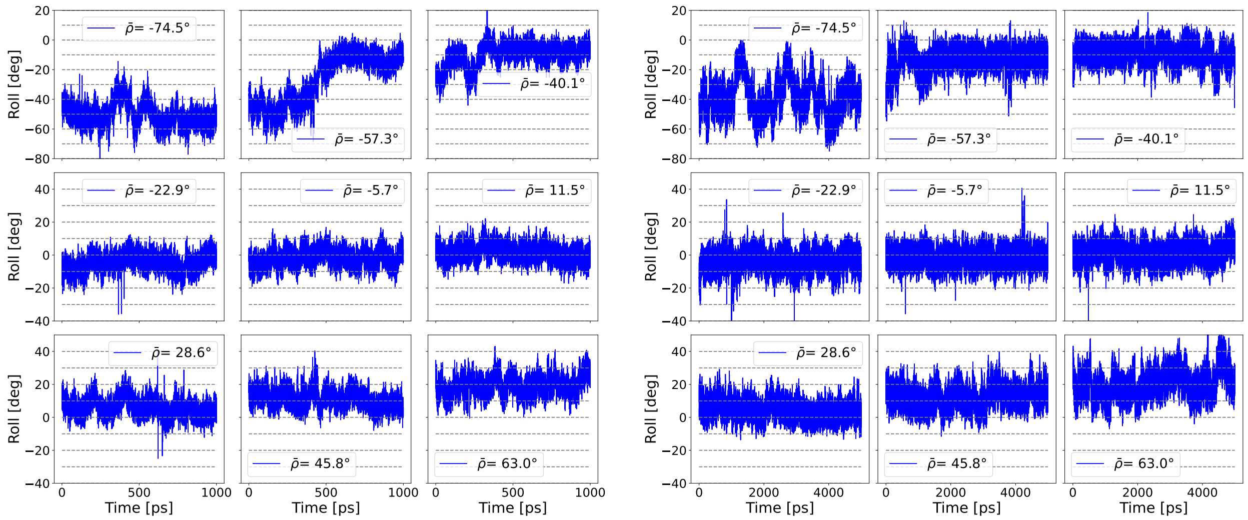

Figure S6: Plots of roll vs. time for 1 ns (left) and 5 ns (right) RBB-NA simulation runs for Sequence 1 with different biases $\bar{\rho}$ and $\gamma = -0.1$. The left figure is the same as Fig. 3 of the main paper.

The plots show the RBB-NA free energies, the average values from the bootstrapping and the 95% CI along lines with slopes $\Omega = -0.1\rho$ for $\rho < 0$ and $\Omega = -0.6\rho$ for $\rho > 0$. Apart from a slight deviations of the RBB-NA data from the bootstrapping mean for Seq. 2 at high $|\rho|$, the other data show very good convergence and the 95% CI is small which is due to the large statistical sampling.

6 Anharmonic Model

We discussed in the Results Section of the main text the analysis of the free energy of Sequence 4 beyond the harmonic approximation. Here, we present the analysis within the same model, described by Eq. 6 of the main text for Sequences 1, 2 and 3. The anharmonic model fits reasonable well the other sequences as well, although the quality is slightly worse for Seq. 1. Table S1 compares the fitted parameters for the four sequences. As already pointed out for Seq. 4 in the main text, one notices a large contribution from a $\sim \Omega^3$ term as opposed to a weak $\sim \rho^3$ ($C_3 \gg A_3^\rho$). The cubic terms B and D, as well as the quartic term A_4^ρ are negative in all sequences analyzed. We note that the sign of C_4 and H however do change for the different sequences.

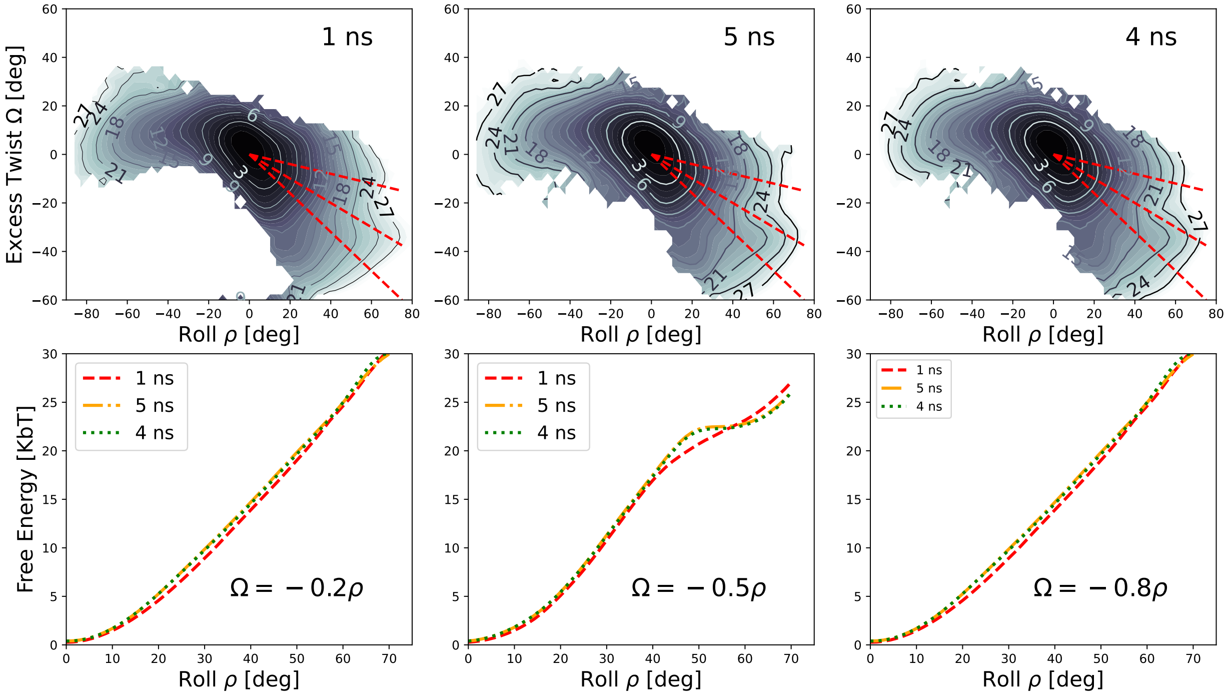

Figure S7: Top row: Comparison of contour plots of the free energy landscape for Sequence 2 (kink in IHF-DNA binding) obtained by the RBB-NA algorithm with samplings of 1 ns, on the left, 5 ns in the center, and the last 4 ns of the 5 ns simulation on the right. The free energies are given in units of k_BT and the contour lines correspond to equal free energy levels, shown every 3 k_BT and up to $27k_BT$. Bottom row: Free energies plotted along three dashed red lines show in the top row graphs corresponding to $\Omega = -0.2\rho$, $\Omega = -0.5\rho$, and $\Omega = -0.8\rho$. The data show good overlap, with deviations occurring for just one inclination for high values in $\rho > 50°$. The good qualitative agreement between the three indicates that the 1 ns simulations provide an adequate sampling of the equilibrium free energies.

To quantify the goodness of fit we added Table S2 which reports the adjusted R^2 value. The adjustment penalizes the addition of fitting parameters that do not significantly improve the model's predictive power. To fit n measurement with p parameters this is defined as:

$$\mathrm{Adj}R^2 = 1 - (1 - R^2) \cdot \frac{n-1}{n-p-1} \tag{1}$$

where R^2 follows the usual definition.

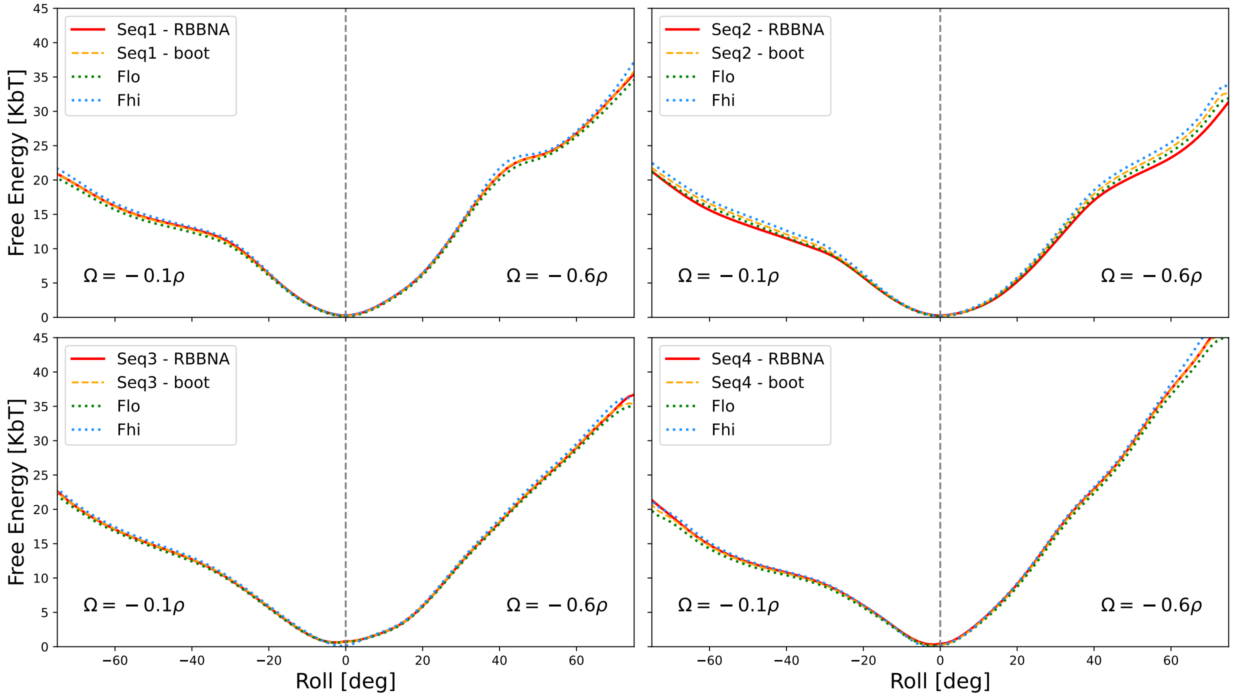

Figure S8: Comparisons of free energy results obtained for RBB-NA simulations (solid red line) and using the bootstrap analysis. The figure shows one-dimensional plots for sequence 1, 2, 3 and 4. Block bootstrapping was performed to them with 100 replicas. The free energy is plotted along the lines with slopes $\Omega = -0.1\rho$ for $\rho < 0$ and $\Omega = -0.6\rho$ for $\rho > 0$. Dashed orange line represents the mean free anergy calculated and Fhi and Flo, the two limits of the 95% CI.

7 Estimating kinks formation time

On the basis of the calculated landscapes we can provide a rough estimate of the time to form a kink. To do that we use a one dimensional Langevin dynamics model with white noise for a reaction coordinate $\alpha(t)$ which will be suitable a combination of roll and twist. These coordinate will be subject to a harmonic force

$$\gamma\dot{\alpha} = -K\alpha + f_R \tag{2}$$

with f_R random uncorrelated noise $\langle f_R(t)f_R(t')\rangle = 2\gamma k_B T\delta(t-t')$. This is known as Orstein-Uhlenbeck model. Solving for the mean-squared displacement one finds

$$\langle\Delta\alpha^2(t)\rangle \equiv \left\langle(\alpha(t)-\alpha(0))^2\right\rangle = \langle\Delta\alpha^2\rangle_{\rm eq}\left(1-e^{-t/\tau}\right) \tag{3}$$

Table S1: Comparisons of fitted parameters for sequences 1,2, 3 and 4. The three coefficients A_2^ρ, C_2 and G are obtained by fitting the harmonic model (first column) within the range $\Delta F \leq 3k_BT$. These values are then kept fixed when fitting the cubic and quartic models. The latter two models are fitted over the broader range $\Delta F \leq 7k_B$. For each sequence, the second column reports coefficients derived using the cubic terms of the anharmonic model (Eq. 6 in the main text), while the third column shows results from fitting the full model of Eq. 6. Quadratic coefficients (A_2^ρ, C_2, G) are multiplied by a factor 10^{-2}, cubic coefficients (A_3^ρ, C_3, B, D) by a factor 10^{-4} and quartic coefficients (A_4^ρ, C_4, H) by a factor 10^{-5}. The data are in dimensionless units as they are obtained by fitting angles in degrees.

	**Seq 1**			**Seq 2**			**Seq 3**			**Seq 4**		
	2nd	3rd	4th	2nd	3rd	4th	2nd	3rd	4th	2nd	3rd	4th
$\boldsymbol{A_2^\rho}$	4.13	4.13	4.13	3.35	3.35	3.35	4.63	4.63	4.63	5.61	5.61	5.61
$\boldsymbol{C_2}$	3.88	3.88	3.88	3.88	3.88	3.88	6.17	6.17	6.17	9.65	9.65	9.65
**G**	1.59	1.59	1.59	1.75	1.75	1.75	3.19	3.19	3.19	1.57	1.57	1.57
$\boldsymbol{A_3^\rho}$	/	0.88	0.84	/	-0.93	-0.68	/	0.72	0.31	/	3.10	0.19
$\boldsymbol{C_3}$	/	2.49	5.62	/	4.22	6.74	/	5.34	5.74	/	12.59	10.52
**B**	/	1.09	-0.60	/	-5.16	-5.00	/	-0.43	-0.73	/	-3.29	-2.49
**D**	/	-3.00	-4.53	/	-0.82	-2.26	/	-0.58	-4.14	/	-16.7	-1.6
$\boldsymbol{A_4^\rho}$	/	/	-2.02	/	/	-1.30	/	/	-1.95	/	/	-4.29
$\boldsymbol{C_4}$	/	/	1.63	/	/	1.08	/	/	-0.30	/	/	-4.26
**H**	/	/	2.54	/	/	3.56	/	/	7.52	/	/	-8.73

Table S2: Calculation of R^2 adjusted for the models proposed. We considered two fitting ranges: $\Delta F \leq 3k_BT$ and $\Delta F \leq 7k_BT$, applied to the three models analyzed: the harmonic model, the the 3rd order model and the full anharmonic model (Eq. 6 of the main text).

		**Harm.Mod.**	**Anh.Mod.** (3rd order)	**Anh.Mod.** (full)
$3k_BT$	Seq 1	0.93	0.93	0.92
	Seq 2	0.91	0.94	0.92
	Seq 3	0.91	0.95	0.97
	Seq 4	0.98	0.98	0.97
$7k_BT$	Seq 1	0.81	0.88	0.95
	Seq 2	0.80	0.89	0.98
	Seq 3	0.86	0.93	0.99
	Seq 4	0.58	0.71	0.99

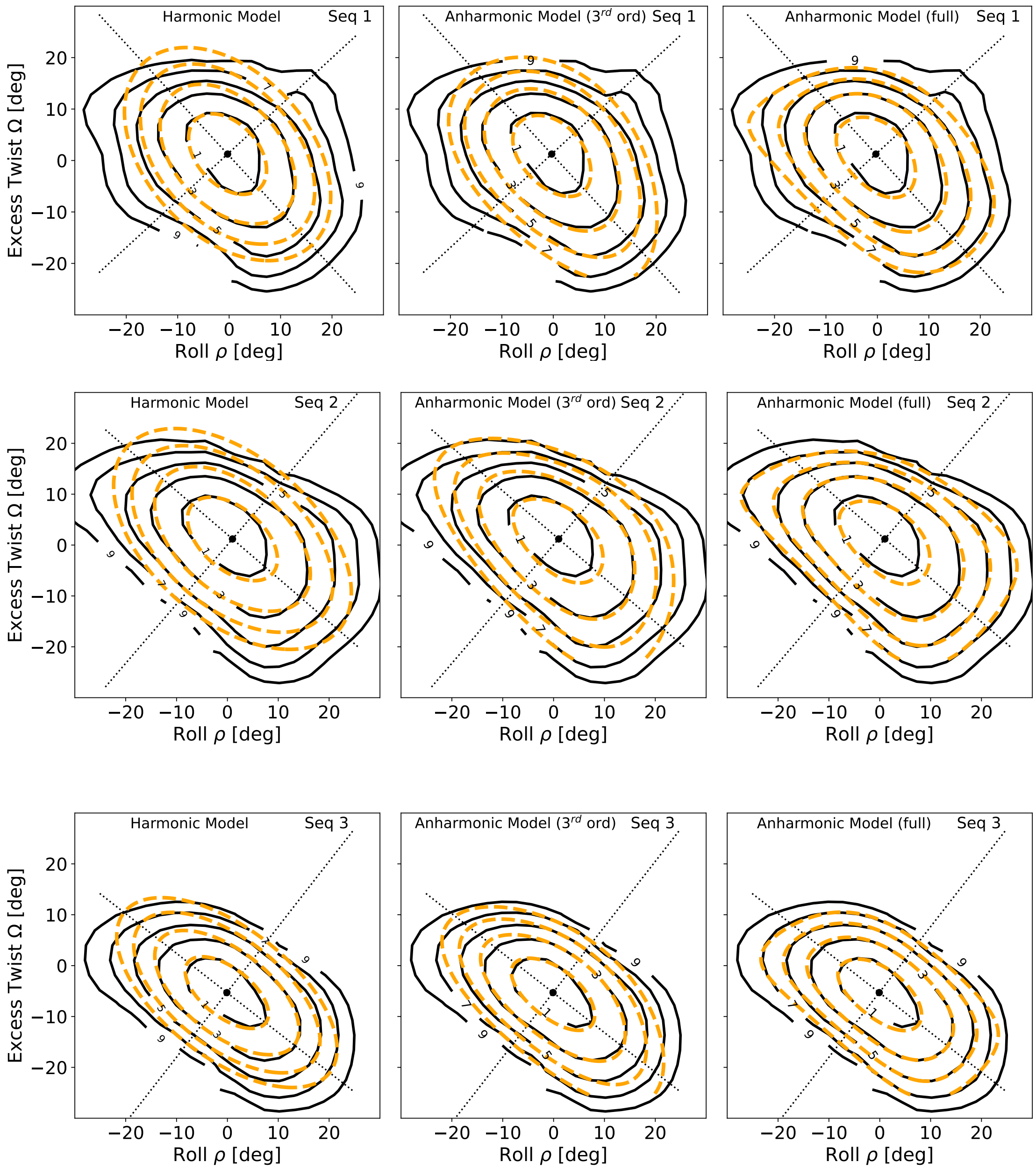

Figure S9: Fitted free energy landscape for sequence 1, 2, 3 (from the top to the bottom). The solid black lines are the all-atom RBB-NA data, while the dashed orange lines are the model. On the left, harmonic model (Eq. 3 of the main text, neglecting tilt) obtained fitting the all-atom data for $\Delta F \leq 3k_BT$. In the middle, anharmonic model (cubic terms of Eq. 6 in the main text) for $\Delta F \leq 7K_BT$. On the right side the fit to the anharmonic model (Eq. 6 of the main text) for $\Delta F \leq 7K_BT$. In black dashed lines the axis of the ellipses of the harmonic model

.

where $\langle \Delta\alpha^2 \rangle_{\rm eq}$ is the equilibrium variance and the relaxation time is $\tau = \gamma/K$. The MSD starts from $\langle \Delta\alpha^2 \rangle(0) = 0$ and converges to the equilibrium values at times $t \gg \tau$.

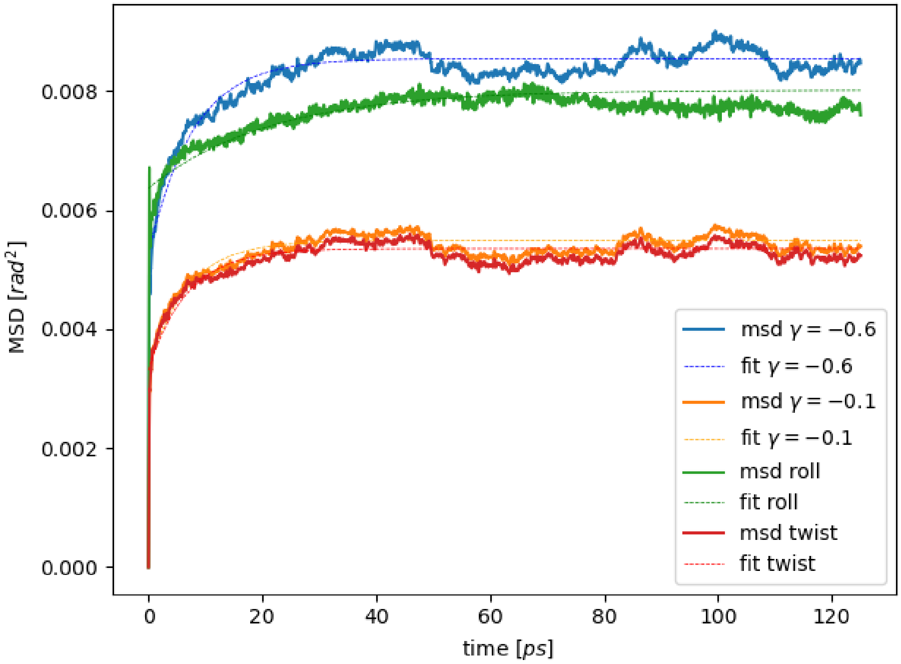

Figure S10: Plots of $\langle \Delta\alpha^2(t) \rangle$ for a few definitions of α along lines of slope $\gamma = -0.1, -0.6$ and for $\alpha = \rho$ and $\alpha = \Omega$. Solid lines are simulation data and dotted lines are fit to Eq. (3). The data give a characteristic equilibration time of $\tau \approx 10$ ns.

Figure S10 shows plots of MSD for different definitions van α (see caption). We estimate from the data an average $\tau \approx 10$ ps $= 10^{-11}$ s. The total time reaching a kinked state can be estimated as

$$T_{\text{kink}} \approx \tau \exp(\Delta F_{\text{kink}}/k_B T) \tag{4}$$

using a free energy barrier model. A barrier of $\Delta F_{\text{kink}} = 20$ $k_B T$ would lead to $T_{\text{kink}} \approx 5\,\text{ms}$. An increase of 3 $k_B T$ corresponds to a factor $e^3 \approx 20$. For the free energies in the range $20 - 26$ $k_B T$ the time scale is from a few milliseconds to a few seconds.

8 Analysis of base-pairing disruptions

Forcing the system into extreme deformations with high twist and roll can disrupt base pairing in the DNA double helix in a RBB-NA simulation. Figure S11 shows two snapshots of biased RBB-NA simulations with a) intact base pairing and b) broken hydrogen bonding leading to base flipping. In the calculation of the free energy landscapes of Fig. 5 of the main

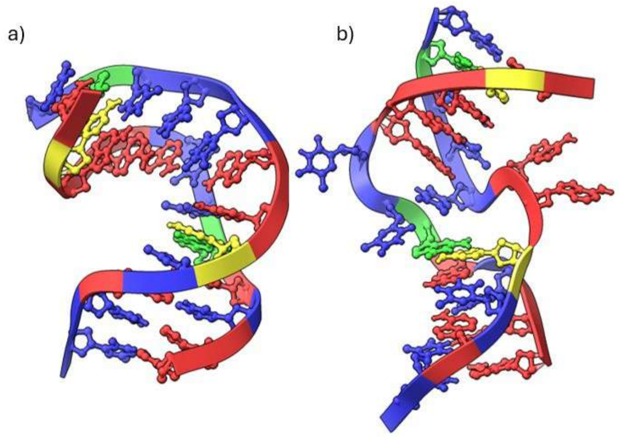

Figure S11: Comparison between snapshots of RBB-NA simulation runs for Sequence 1 biased to (a) $\bar{\rho} = 85.94°$ (1.5 rad), $\gamma = -0.9$ and (b) $\bar{\rho} = 80.21°$ (1.4 rad), $\gamma = -0.9$. In the case (a) the sequence can withstand the strong bias without breaking the hydrogen bonds. In the case (b) the bias leads to hydrogen bond breakage and four bases simultaneously flipping out. Configurations of the type (b) are excluded from the WHAM analysis as our focus is to determine free energies for configurations which preserve base pairing.

text configurations with broken hydrogen bonds were excluded from the analysis. This was done as follows. During the simulation runs four additional intra base pair parameters Stagger, Shear, Stretch and Opening were monitored. Using cgDNA+ we computed the standard deviation of each parameter for every sequence as a reference value. At the end of the simulation run, using RBB-NA we then computed the mean of the parameters. For a Gaussian distribution, deviations exceeding four standard deviations correspond to a probability equal to $P(|x| > 4\sigma) = 1 - \mathrm{Erf}(2\sqrt{2}) = 6.33 \cdot 10^{-5}$, where $\mathrm{Erf}(x)$ is the error function. The free energy cost of such conformations can be estimated from $-k_B T \log P(|x| > 4\sigma) \approx 10\, k_B T$.

We then analyzed the frequency of breakage events and found that they generally occur at extreme values of roll, particularly in the intervals $\bar{\rho} \lesssim -45°$ and $\bar{\rho} \gtrsim 45°$ (0.8 rad). The side on which breakage occurs depends both on the sequence and on the value of γ. For sequence 4 and sequence 6, the majority of breakages is for $\rho < 0$ whereas for the others sequences, breakages are more evenly distributed between positive and negative roll. When considering the contribution of γ across all sequences, we noted that for small Twist (e.g. $\gamma = -0.1$), most breakages are localized on the positive roll (10 in positive versus 5 in the negative). However, as the Twist increases (e.g. $\gamma = -0.9$) the total number of breakages

rises significantly to 48, with the majority (37) laying on the negative side of roll and only 11 for positive roll.

Figure S12 shows as red crosses the values of the bias of roll and twist in which we observe the disruption. Most of these crosses fall outside the range of ρ and Ω in which the free energy is evaluated. In a few cases they are inside the free energy domain. This happens because the criterion to flag a base-pair disruption event is based on fluctuations exceeding some threshold value (see main text). It may happen that for some given bias $\bar{\rho}$, $\bar{\Omega}$ this threshold is passed and the simulation gets flagged as base-pair disruption event. Then, when the bias is slightly exceeded, it may happen that no base-pair disruptions are detected and the free energy gets evaluated. The appearance of a few isolated crosses within the free energy domain in Fig. S12 is due to some occasional false positive detection. We note that, in general, overtwisting leads to much more frequent basepairing disruptions than undertwisting. Some sequences, as Seq. 4 and Seq. 6, can tolerate a large degree of undertwisting while maintaining intact base-pairing.

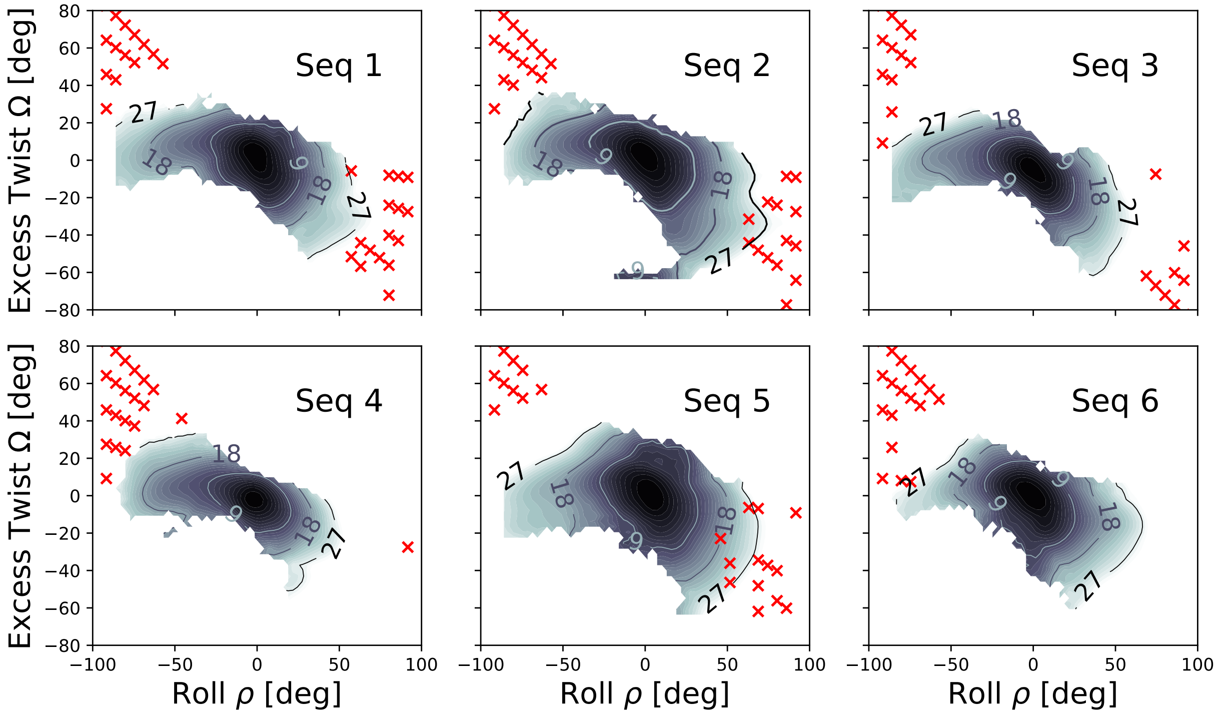

Figure S12: Contour plots of the free energy landscapes, for the six sequences studied, obtained by the RBB-NA algorithm via umbrella sampling and WHAM analysis. The free energies are given in units of $k_B T$ and the contour lines correspond to equal free energy levels, shown every 9 $k_B T$ and up to 27 $k_B T$. The focus is on the configurations where base disruption was observed, indicated by the red crosses in the figure.

References

Mitchell, J et al (2011) "Atomistic simulations reveal bubbles, kinks and wrinkles in supercoiled DNA". Nucl. Acids Res. **39**, 3928–3938.

Mouw, K. W. and Rice, P. A. (2007) "Shaping the Borrelia burgdorferi genome: crystal structure and binding properties of the DNA-bending protein Hbb". *Mol. Microbiol.*, **63**, 1319–1330.

Olson, W. K., et al. (1998). "DNA sequence-dependent deformability deduced from protein–DNA crystal complexes". *Proc. Natl. Acad. Sci.*, **95**, 11163–11168.

Schultz, S., et al. (1991). "Crystal structure of a CAP- DNA complex: the DNA is bent by 90 degrees". *Science*, **253**, 1001–1007.

Lankaš, F., et al. (2006). "Kinking Occurs during Molecular Dynamics Simulations of Small DNA Minicircles". *Structure*, **14**, 1527–1534.

Vasudevan, D., et al. (2010). "Crystal Structures of nucleosome core particles containing the '601' strong positioning sequence". *Journal of Molecular Biology* **403.1**, 1-10.
